# A direct link between MITF, innate immunity, and hair graying

**DOI:** 10.1371/journal.pbio.2003648

**Published:** 2018-05-03

**Authors:** Melissa L. Harris, Temesgen D. Fufa, Joseph W. Palmer, Sandeep S. Joshi, Denise M. Larson, Arturo Incao, Derek E. Gildea, Niraj S. Trivedi, Autumne N. Lee, Chi-Ping Day, Helen T. Michael, Thomas J. Hornyak, Glenn Merlino, NISC Comparative Sequencing Program, William J. Pavan

**Affiliations:** 1 Department of Biology, University of Alabama at Birmingham, Birmingham, Alabama, United States of America; 2 Genetic Disease Research Branch, National Human Genome Research Institute, National Institutes of Health, Bethesda, Maryland, United States of America; 3 Department of Biochemistry and Molecular Biology, University of Maryland, School of Medicine, Baltimore, Maryland, United States of America; 4 Computational and Statistical Genomics Branch, National Human Genome Research Institute, National Institutes of Health, Bethesda, Maryland, United States of America; 5 Laboratory of Cancer Biology and Genetics, Center for Cancer Research, National Cancer Institute, National Institutes of Health, Bethesda, Maryland, United States of America; 6 Research & Development Service, VA Maryland Health Care System, Baltimore, Maryland, United States of America; University of Michigan, United States of America

## Abstract

Melanocyte stem cells (McSCs) and mouse models of hair graying serve as useful systems to uncover mechanisms involved in stem cell self-renewal and the maintenance of regenerating tissues. Interested in assessing genetic variants that influence McSC maintenance, we found previously that heterozygosity for the melanogenesis associated transcription factor, *Mitf*, exacerbates McSC differentiation and hair graying in mice that are predisposed for this phenotype. Based on transcriptome and molecular analyses of *Mitf*^*mi-vga9/+*^ mice, we report a novel role for MITF in the regulation of systemic innate immune gene expression. We also demonstrate that the viral mimic poly(I:C) is sufficient to expose genetic susceptibility to hair graying. These observations point to a critical suppressor of innate immunity, the consequences of innate immune dysregulation on pigmentation, both of which may have implications in the autoimmune, depigmenting disease, vitiligo.

## Author summary

Hair pigmentation over the course of a lifetime depends on melanocyte stem cells that reside in the hair follicle. As old hairs fall out and new hairs grow in, melanocyte stem cells serve as a reservoir for the melanocytes that produce the pigment that gives hair its visible color. The loss of these stem cells leads to the growth of nonpigmented, or gray, hairs. Evaluating mouse models of hair graying can reveal key aspects of melanocyte stem cell biology. Using this approach, we discovered a novel role for the melanogenesis associated transcription factor, MITF, in repressing the expression of innate immune genes within cells of the melanocyte lineage. The importance of this repression is revealed in animals that have a predisposition for hair graying. In these animals, artificial elevation of the innate immune response, either through a genetic mechanism or via exposure to viral mimic, results in significant melanocyte and melanocyte stem cell loss and leads to the production of an increased number of gray hairs. These observations highlight the negative effects of innate immune activation on melanocyte and melanocyte stem cell physiology and suggest a connection between viral infection and hair graying.

## Introduction

In the 1980s, a handful of studies reported that exposure to murine leukemia virus (MuLV), either at mid-gestation or perinatally, is sufficient to drive premature hair graying in mice [1–3]. Early infection with MuLV does not lead to immediate loss of hair pigmentation and instead produces an adult-onset, progressive hypopigmentation phenotype, suggestive of a failure in melanocyte lineage regeneration. These observations suggest a role for innate immune activation in adult hypopigmentation disorders, but how this phenomenon is mediated within the postnatal melanocyte lineage remains unresolved. Using approaches to look for genetic modifiers of hair graying in mice and transcriptomic analysis of melanocyte stem cells (McSCs), we identify an exciting and unexpected link between the melanogenesis associated transcription factor, MITF, and the suppression of a type I interferon (IFN) gene signature. This discovery creates a unique opportunity to investigate how innate immune gene expression is regulated in postnatal melanocytes and how its dysregulation affects McSCs and the regeneration of postnatal pigmentation during hair cycling.

During hair growth, McSCs produce the melanocyte progeny that differentiate and deposit melanin into the hair shaft. Mouse models reveal that hair graying, both acute and age related, is frequently preceded by a failure in McSC maintenance or dysregulated generation of melanocyte progeny. Both lead to the production of nonpigmented, or gray, hair shafts. Hair graying can be elicited through a number of mechanisms—disrupting the signaling pathways associated with the Kit receptor, Notch receptor, Endothelin receptor type B, Raf kinase, Transforming growth factor beta, or Wnt [4–11]; loss of anti-apoptotic control [12,13]; melanocyte-specific dysregulation of chromatin remodeling complexes [14]; exposure to genotoxic stress [15,16]; changes in sex determining region Y-box 10 (SOX10) or MITF-mediated transcriptional regulation [13,17]; vitiligo-like T-cell–mediated destruction of melanocytes [18]; and aging itself [13].

In particular, MITF, the gene encoding the micropthalmia-associated transcription factor, is essential at multiple stages of the melanocyte life cycle. Across species, MITF is required for the specification and survival of the melanocyte precursors, or melanoblasts, during early neural crest migration [19–21]. Loss of *Mitf* expression results in the near-complete depletion of embryonic melanoblasts, and this leads to mice born with a fully white coat [22]. During melanogenesis, MITF transcriptionally regulates a number of pigmentation genes involved in the biosynthesis of melanin and the trafficking of melanosomes. Regulation of MITF activity levels also influences the dynamic transition between the states of melanocyte migration, proliferation, and differentiation [23–25].

**Fig 1:**
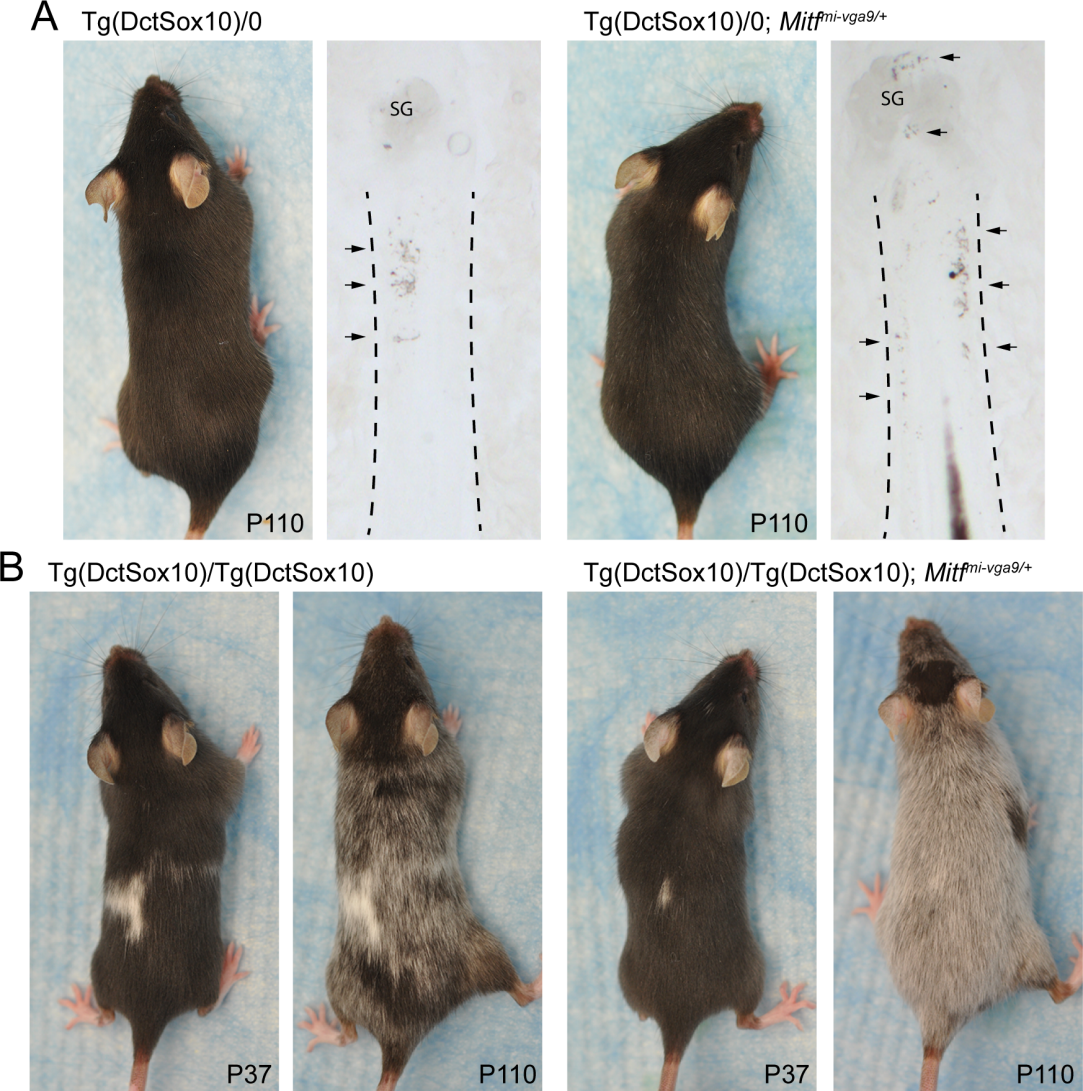
Increased hair graying and McSC differentiation occurs in Tg(Dct-Sox10) animals that are haploinsufficient for *Mitf*^*mi-vga9/+*^. **(A)** Dorsal images of two female littermates taken at P110. At this age, the coat of Tg(Dct-Sox10)/0 mice appears solid black, while the coat of Tg(Dct-Sox10)/0; *Mitf*^*mi-vga9/+*^ mice exhibits a small number of gray hairs along its back. Bright-field images of histological sections of the skins from these mice reveal increased ectopic pigmentation (arrows) within the hair bulge (region between the dotted lines) of hairs from Tg(Dct-Sox10)/0; *Mitf*^*mi-vga9/+*^ animals in comparison to Tg(Dct-Sox10)/0 animals. **(B)** Dorsal images of two female littermates taken at P37 and P110. At P37, Tg(Dct-Sox10)/Tg(Dct-Sox10) mice exhibit a black coat with congenital white spotting on the belly and back. These mice experience progressive hair graying and by P110 exhibit a “salt and pepper” coat color in regions of the back fur that were originally black. In comparison to Tg(Dct-Sox10)/Tg(Dct-Sox10) mice, hair graying in Tg(Dct-Sox10)/Tg(Dct-Sox10); *Mitf*^*mi-vga9/+*^ mice at P110 is more severe, with a majority of the back fur exhibiting gray hairs. The images for this figure were generated in the original study presented in [17]. McSC, melanocyte stem cell; P, postnatal day; SG, sebaceous gland.

Our interest in the role of MITF in hair graying was spurred by our previous observation that a genetic interaction exists between *Mitf* and the *Sox10* transgene, Tg(Dct-Sox10) [17]. Tg(DctSox10) mice conditionally overexpress *Sox10* within the melanocyte lineage, and this leads to the premature differentiation of McSCs, eventual McSC depletion, and progressive hair graying. Because SOX10 transcriptionally activates the *Mitf* gene and the MITF protein promotes melanocyte differentiation, we anticipated that reducing *Mitf* expression might alleviate Tg(DctSox10)-mediated hair graying. We tested this using the *Mitf-*null allele, *Mitf*^*mi-vga9*^, which in the heterozygous state does not lead to apparent McSC dysfunction or hair graying. However, in contrast to our expectations, Tg(Dct-Sox10)/0; *Mitf*^*mi-vga9/+*^ mice exhibit sparsely distributed yet noticeable nonpigmented or “gray” hairs throughout their coat prior to their Tg(Dct-Sox10)/0 littermates (**Fig 1A**). Tg(Dct-Sox10)/0; *Mitf*^*mi-vga9/+*^ mice also show increased differentiation of McSCs, as indicated by excessive ectopic pigmentation within the stem cell niche (hair bulge) of their hair follicles. Intensified adult-onset hair graying associated with *Mitf*^*mi-vga9*^ is particularly evident when the Tg(Dct-Sox10) transgene is homozygous and animals are imaged over time (**Fig 1B**). We interpreted this outcome as contrary to the canonical role of MITF, in which high levels of MITF activity, rather than low, are associated with cell cycle arrest and melanocyte differentiation [26]. This interpretation is reinforced further by the fact that McSCs in *Mitf*^*vit/vit*^ mice, which carry a hypomorphic mutation of *Mitf*, also exhibit premature McSC differentiation and hair graying even in the absence of Tg(Dct-Sox10) [13]. This suggests a novel contribution of MITF to the regulation of McSC maintenance and melanocyte lineage regeneration postnatally.

With this in mind, we performed genomic analysis of wild-type and *Mitf*^*mi-vga9/+*^ McSCs and discovered that haploinsufficiency for *Mitf* results in a marked and chronic up-regulation of a type I IFN gene signature in this stem cell population. To better understand the contribution of innate immune suppression in postnatal melanogenesis, we further validated this immune signature in the skin and melanoblasts (the melanocyte precursors) of *Mitf*^*mi-vga9/+*^ mice. We evaluate the possibility that MITF acts as negative transcriptional regulator of innate immune target genes, and we assess the consequence of innate immune pathway activation on the regeneration of the melanocyte lineage during hair cycling.

## Results

### *Mitf* haploinsufficiency is associated with an up-regulation of a type I IFN–regulated gene expression signature in McSCs and melanoblasts

**Fig 2:**
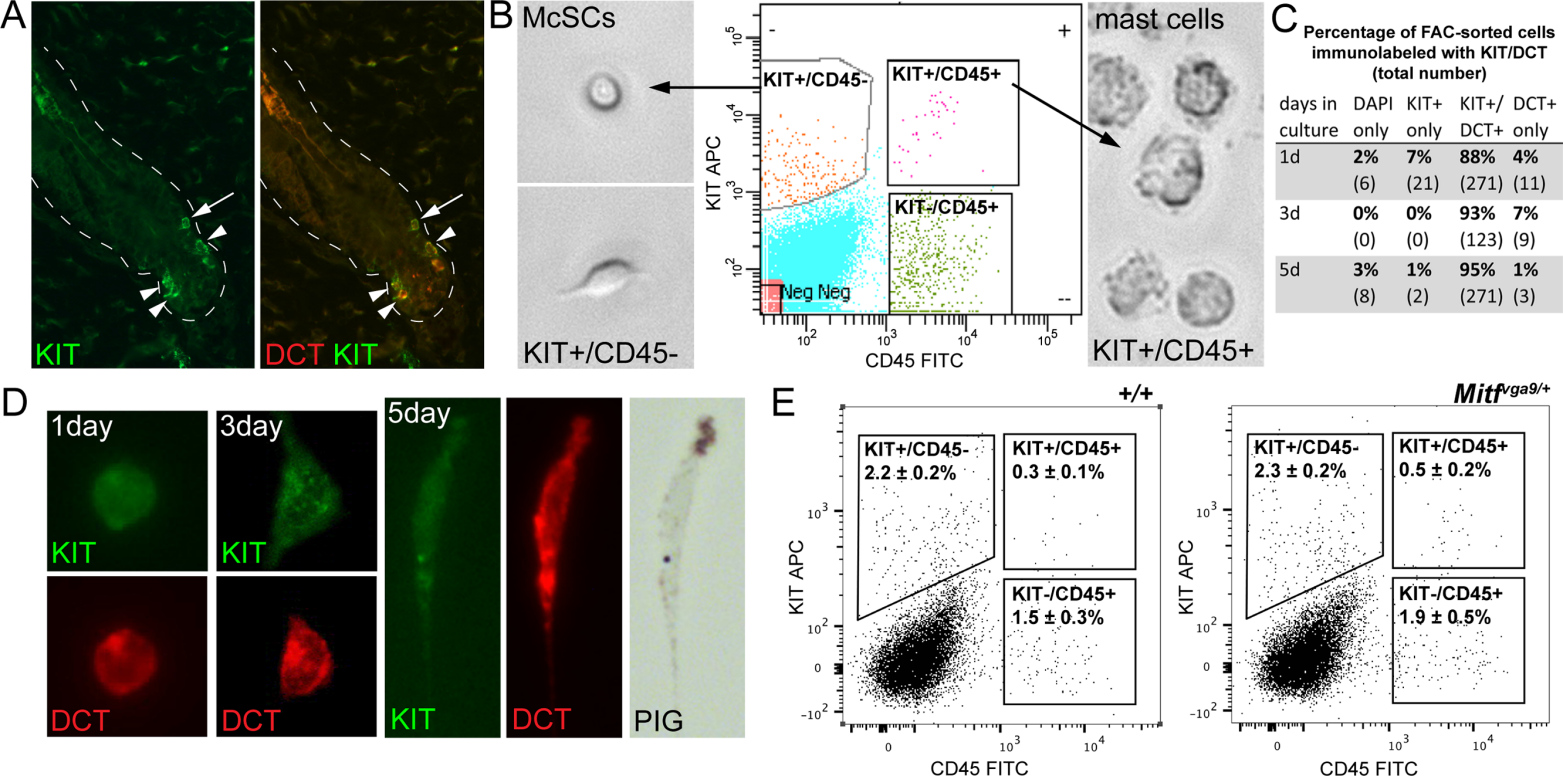
FACS isolation of adult McSCs. **(A)** Immunolabeling for KIT protein in mouse skin at 8 weeks of age. At this time point, the majority of hairs exist in the telogen hair stage, and McSCs that are positive for both KIT (green) and DCT (red) are observed in the hair bulge (arrow) and secondary hair germ (arrowheads). The dotted white lines outline the hair follicles. **(B)** FACS of dermal cells from 8-week-old mice produces two KIT+ populations. McSCs are CD45− and mast cells are CD45+. The FACS gating strategy (center fluorescence plot) used to isolate McSCs successfully separates each of these cell types and is confirmed by visualizing their distinct morphologies; McSCs are small and often bipolar (left phase images), while mast cells are large and rough looking (right phase image). **(C)** KIT+/CD45− cells isolated by FACS were placed in culture and assessed for their expression of KIT and DCT by immunolabeling over a 5-day period. Total cells were identified by the nuclear marker DAPI. The table shows the percentage and total number (in parentheses) of cells exhibiting the indicated staining pattern. This FACS protocol produces a relatively pure McSC population, with >92% of cells being DCT+ 1 day after sorting. **(D)** FACS-isolated KIT+/CD45− McSCs remain positive for the melanocyte markers KIT (green) and DCT (red) and exhibit melanocyte-like traits while in tissue culture. These cells progress from being round at 1 day, to slightly spread at 3 days, to dendritic and pigmented over 5 days. **(E)** Evaluation of the indicated gates (boxes) on FACS fluorescence plots confirms that there is no significant difference by *t* test when comparing the percentage of each cell population between wild-type (left plot) and *Mitf*^*mi-vga9/+*^ (right plot) dermal cell suspensions (KIT+/CD45−, *p* = 0.85; KIT+/CD45+, *p* = 0.14; KIT−/CD45+, *p* = 0.28). Percentages are represented as the mean ± standard deviation, with *n* = 3 sorts per genotype. The raw data used to generate these graphs are available in **S1 Data**. APC, allophycocyanin; CD45, cluster of differentiation 45; DCT, dopachrome tautomerase; FACS, fluorescence-activated cell sorting; FITC, fluorescein; McSC, melanocyte stem cell; *Mitf*, melanogenesis associated transcription factor; Neg, negative; PIG, pigment.

In order to assess the effects of *Mitf*^*mi-vga9/+*^ on gene expression in vivo, McSCs were isolated from the dermis of adult mice at 8 weeks of age. At this time point, hairs across the body of the mouse are synchronized in the hair stage of telogen [27]. During telogen, the hair follicle does not have a hair bulb or differentiated melanocytes, and McSCs are the only melanocytic cells present within the hair. They can be identified by their expression of the melanogenic enzyme dopachrome tautomerase (DCT) and the transmembrane receptor KIT, and they are observed in the hair bulge (the upper region of the telogen-stage hair that lies at the insertion point of the arrector pili muscle) and secondary hair germ (the lower region of the telogen-stage hair nearest the dermal papilla; **Fig 2A**). In order to isolate these McSCs from telogen hairs, the dermis of *Mitf*^*mi-vga9/+*^ and wild-type mice was dissociated and immunolabeled with two cell-surface markers, KIT and cluster of differentiation 45 (CD45). Fluorescence-activated cell sorting (FACS) was then used to distinguish McSCs (KIT+/CD45−) from mast cells (KIT+/CD45+; **Fig 2B**). When assessed in vitro 1, 3, or 5 days after sorting, greater than 92% of the KIT+/CD45− population of cells express the melanocytic protein DCT and begin to produce pigment at 5 days (**Fig 2C and 2D**). Additionally, because MITF is a known transcriptional regulator of the *Kit* gene [20,28], we compared the population percentages within each FACS gate between the wild-type and *Mitf*^*mi-vga9/+*^ dermal cell suspensions. No significant difference is observed in the percent of KIT+/CD45− dermal cells between wild-type and *Mitf*^*mi-vga9/+*^ animals, suggesting that the *Mitf*^*mi-vga9*^ mutation does not change the ability of this FACS strategy to identify the McSC population (**Fig 2E**). This suggests that this sorting strategy is adequate to produce a highly enriched pool of McSCs for transcriptomic analysis. RNA isolated from the KIT+/CD45− McSC populations obtained from the dermis of wild-type and *Mitf*^*mi-vga9/+*^ mice was then subjected to RNA sequencing (RNA-seq).

**Fig 3:**
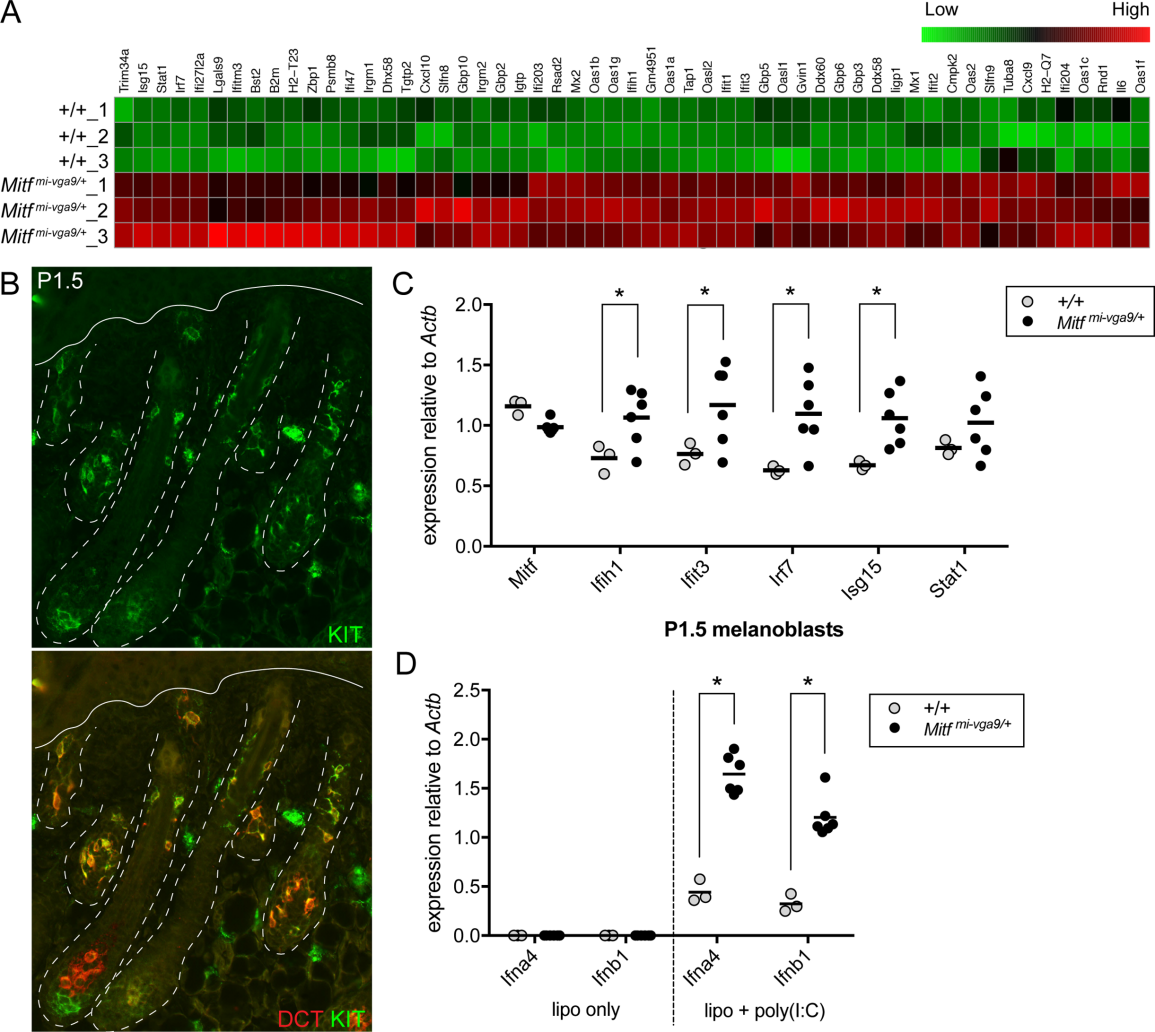
*Mitf*^*mi-vga9/+*^ McSCs and melanoblasts exhibit an elevated type I interferon gene expression signature and *Mitf*^*mi-vga9/+*^ melanoblasts show increased sensitivity to viral mimic. **(A)** Heatmap of scaled and clustered, rlog-transformed read count values obtained from RNA-seq analysis of *Mitf*^*mi-vga9/+*^ and wild-type McSCs. Fifty-five of the four hundred eleven genes that demonstrated a statistically significant, greater than 1.5-fold increase in expression in *Mitf*^*mi-vga9/+*^ McSCs over wild-type McSCs participate in innate immune signaling and are presented here (*p*^adjusted^ < 0.05, Benjamini-Hochberg adjusted *p*-value). **(B)** Immunolabeling for KIT protein in mouse skin at P1.5. At this time point, KIT+ (green), DCT+ (red) melanoblasts are observed migrating into the developing hair follicles. The dotted white lines outline the hair follicles and the solid line indicates the position of the epidermis. **(C)** qRT-PCR analysis of *Mitf* and ISG expression (*Ifih1*, *Ifit3*, *Irf7*, *Isg15*, and *Stat1*) in primary melanoblasts isolated from *Mitf*^*mi-vga9/+*^ and wild-type littermate pups at P1.5. Each circle indicates the expression of cells from an individual primary melanoblast cell line generated from an individual pup. The horizontal bars represent the mean, and the asterisks indicate gene expression changes with a *q*-value of <0.05 using the two-stage linear step-up procedure of Benjamini, Krieger, and Yekutieli, with *Q* = 5%. **(D)** qRT-PCR analysis of type I interferon gene expression (*Ifna4* and *Ifnb1*) in primary melanoblast cell lines (isolated as described in [C]). Melanoblast cell lines were treated for 9 hours with lipofectamine only (lipo only) or lipofectamine and poly(I:C) (lipo + poly(I:C)). The horizontal bars represent the mean, and the asterisks indicate gene expression changes with a *q*-value of <0.05 using the two-stage linear step-up procedure of Benjamini, Krieger, and Yekutieli, with *Q* = 5%. The data presented here are representative of two independent experiments testing poly(I:C) treatment of primary melanoblast cell lines at two different cell passages. The raw data used to generate the graphs in (C) and (D) are available in S1 Data. DCT, dopachrome tautomerase; ISG, IFN-stimulated gene; lipo + poly(I:C), lipofectamine and poly(I:C); lipo only, lipofectamine only; *Mitf*, melanogenesis associated transcription factor; McSC, melanocyte stem cell; P, postnatal day; qRT-PCR, quantitative reverse transcriptase polymerase chain reaction; RNA-seq, RNA sequencing.

Using a 1.5-fold cutoff, differential gene expression analysis of the RNA-seq reads obtained from *Mitf*^*mi-vga9/+*^ and wild-type McSCs demonstrated that, as expected, *Mitf*^*mi-vga9/+*^ McSCs exhibit the reduced expression of pigmentation-related genes known to be positively regulated by MITF (**S2 Data**). This includes *Dct*, *Gpnmb*, *Gpr143*, *Irf4*, *Kit*, *Mlana*, *Met*, *Rab27a*, *Slc45a2*, *Tbx2*, and *Tyr*. However, this analysis also uncovered an unanticipated enrichment of DEGs up-regulated in *Mitf*^*mi-vga9/+*^ McSCs that are involved in innate immunity (**S2 Data**). Out of the 411 genes with a greater than 1.5-fold up-regulation in *Mitf*^*mi-vga9/+*^ cells, at least 55 are known for their involvement in type I innate immune signaling (DAVID Functional Annotation Tool [29,30] and interferome.org [31]). These include cytoplasmic pattern recognition receptors (PRRs; *Ddx58*, *Ifih1*), transcriptional regulators (*Irf7*, *Stat1*), and IFN-stimulated genes (ISGs) that execute the antiviral response (*Ifit1*, *Ifit3*, *Isg15*, *Mx1*, *Oas1a*; **Fig 3A, S3 Data**). Up-regulation of this particular subset of genes is known as the “IFN signature” and is often associated with viral infection and autoimmune disorders [32]. To rule out the possibility that this ISG signature was due to a microbial infection of our *Mitf*^*mi-vga9*^ line at the time that McSCs were harvested for FACS, we assessed ISG expression by quantitative reverse transcriptase polymerase chain reaction (qRT-PCR) in skins obtained from mice at postnatal day 32 (P32) that were housed in an unrelated facility and that have been genetically isolated from our *Mitf*^*mi-vga9*^ line for several generations. Consistent with our RNA-seq data, skins from these *Mitf*^*mi-vga9/+*^ mice show clear up-regulation of the five ISGs evaluated, *Ifih1*, *Ifit3*, *Irf7*, *Isg15*, and *Stat1*, in comparison to animals that do not carry this mutation (**S1 Fig)**.

Type I innate immune signaling is comprised of a two-part signaling cascade. First, in an infected cell, viral DNA or RNA is recognized by PRRs, and downstream signaling results in the phosphorylation of interferon regulatory factors (IRFs). Active IRFs enter the nucleus and transcriptionally up-regulate the type I IFN genes to produce the chemokines IFN-α and -β. Via autocrine and paracrine signaling, IFNs bind IFN receptors and activate a second signaling cascade that results in a broad and robust ISG program. These ISGs mediate the antiviral response in both the infected and neighboring cells [33–36]. Based on this, we reasoned that *Mitf*^*mi-vga9/+*^ McSCs should more readily up-regulate *Ifn* gene expression in response to virus in comparison to wild-type McSCs if the IFN signature associated with *Mitf*^*mi-vga9*^ is indeed genuine. To test this, viral infection can be mimicked by delivering synthetic dsRNA into the cell cytoplasm via lipofectamine-based transfection. Polyinosinic:polycytidylic acid (poly(I:C)) is one type of dsRNA, an agonist for the cytoplasmic PRR, melanoma differentiation-associated gene 5 (MDA5, encoded by the *Ifih1* gene), and is sufficient to mediate type I IFN responses in vitro and in vivo [37].

Techniques for expanding McSCs in culture for in vitro analysis are not well established, so we instead evaluated primary melanoblasts for their response to viral mimic. Primary melanoblasts refer to the highly proliferative, KIT+, melanocyte precursors that invade the developing hair follicle and give rise to either McSCs or differentiated melanocytes during perinatal hair morphogenesis (**Fig 3B**). Using the same FACS strategy as presented above (**Fig 2B)**, KIT+/CD45− primary melanoblasts can be isolated from the whole skin of pups on P1.5 and passaged readily in tissue culture. Comparing cells isolated from wild-type and *Mitf*^*mi-vga9/+*^ littermates, we first confirmed that *Mitf*^*mi-vga9/+*^ melanoblasts exhibit an IFN signature similar to adult McSCs. By qRT-PCR we assessed the ISGs *Ifih1*, *Ifit3*, *Irf7*, *Isg15*, and *Stat1* and demonstrated that *Mitf*^*mi-vga9/+*^ melanoblasts show a significant up-regulation of several of these ISGs, namely *Ifih1*, *Ifit3*, *Irf7*, and *Isg15* (**Fig 3C**). Despite this IFN signature, basal *Ifn* gene expression in *Mitf*^*mi-vga9/+*^ and wild-type melanoblasts is equivalent and nearly undetectable (**Fig 3D,** lipo only). However, transient transfection of these melanoblasts with poly(I:C) induces the expression of *Ifna4* and *Ifnb1* in both cell types, with *Mitf*^*mi-vga9/+*^ melanoblasts expressing 2- to 3-fold higher *Ifn* mRNA levels than wild-type melanoblasts (**Fig 3D,** lipo + poly(I:C)). ISG expression in perinatal melanoblasts demonstrates that the IFN signature observed in *Mitf*^*mi-vga9/+*^ animals is not exclusive to the McSC or the adult time point. The similarity in basal *Ifn* expression between wild-type and *Mitf*^*mi-vga9/+*^ melanoblasts also suggests that the IFN signature observed in *Mitf*^*mi-vga9/+*^ melanoblasts is not due to the constant production of IFN by melanoblasts. Furthermore, the enhanced *Ifn* expression exhibited by *Mitf*^*mi-vga9/+*^ melanoblasts after exposure to viral mimic suggests that haploinsufficiency for *Mitf* primes these melanoblasts for the antiviral response.

### MITF binds the promotor of several ISGs and transcriptionally represses ISG expression in vitro

**Fig 4:**
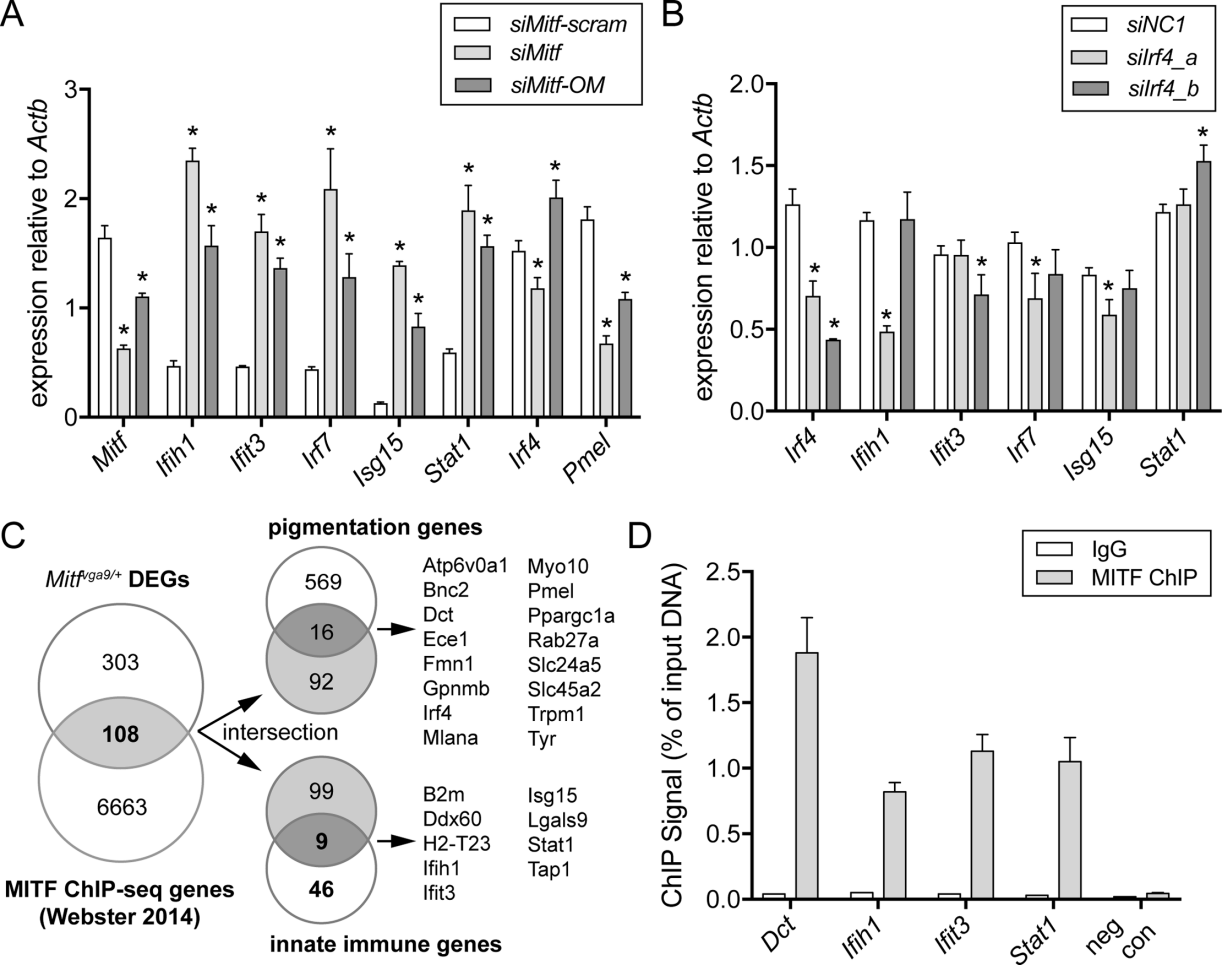
MITF binds to the promoter of innate immune target genes and acts as a transcriptional repressor. **(A, B)** Relative gene expression in melan-a cells 48 hours after transfection with siRNA. In **(A)**, cells were transfected with siRNAs targeting *Mitf* (*siMitf* and *siMitf-OM*) or an analogous scrambled control siRNA (*siMitf-scram*). In **(B)**, cells were transfected with one of two siRNAs targeting *Irf4* (*siIrf4_a*, *siIrf4_b*) or *siNC1*. The bars represent the mean ± standard deviation, and the asterisks indicate gene expression changes with a *q*-value of <0.05 using the two-stage linear step-up procedure of Benjamini, Krieger, and Yekutieli, with *Q* = 5%. Data presented in A and B are representative of two independent knockdown experiments performed in melan-a cells at two different cell passages. **(C)** Venn diagrams demonstrating the intersection of gene lists. The left diagram represents the genes exhibiting a 1.5-fold or greater difference in expression in *Mitf*^*mi-vga9/+*^ over wild-type McSCs (*Mitf*^*mi-vga9/+*^DEGs, top circle) overlapped with the list of genes associated with MITF ChIP-seq peaks reported by Webster et al. 2014 (MITF ChIP-seq genes, bottom circle) [41] and are considered potential direct targets of MITF. These 108 direct targets are provided in **S4 Data**. Direct target genes were further identified by comparing them with a list of known pigmentation genes in the upper right diagram and with the list of 55 innate immune-related genes up-regulated in *Mitf*^*mi-vga9/+*^ McSCs (as defined in Fig 3A) in the lower right diagram. **(D)** MITF ChIP-qPCR performed in melan-a cells and assayed for enrichment at the indicated gene loci (*Dct*, *Ifih1*, *Ifit3*, *Stat1*). A centromeric DNA sequence was amplified as a negative control (neg con). Error bars indicate mean ± standard deviation of two independent ChIP pulldowns. The raw data used to generate the graphs in (A), (B), and (D) are available in **S1 Data**. *Actb*, actin beta; ChIP, chromatin immunoprecipitation; ChIP-qPCR, chromatin immunoprecipitation quantitative polymerase chain reaction; ChIP-seq, chromatin immunoprecipitation sequencing; DEG, differentially expressed gene; IgG, immunoglobulin G; McSC, melanocyte stem cell; MITF, melanogenesis associated transcription factor; neg con, negative control; *siNC1*, nontargeting control siRNA; siRNA, small interfering RNA.

MITF is a transcription factor that promotes the direct and indirect regulation of a number of genes essential for melanocyte development, differentiation, proliferation, and survival [38]. Thus, we investigated whether the IFN signature observed in *Mitf*^*mi-vga9/+*^ McSCs and melanoblasts may be dependent on MITF’s role as a transcription factor. To determine whether reduction of *Mitf* is sufficient to lead to a direct change in ISG expression autonomous to the melanocyte, we knocked down *Mitf* in the immortalized mouse melanocyte cell line melan-a using a small interfering RNA (siRNA) approach. Intracellular delivery of siRNAs can elicit a nonspecific innate immune response that can be mitigated by incorporating 2′-*O*-methyl-uridine or guanosine nucleosides into at least one strand of the siRNA duplex [39]. Thus, we generated two siRNAs—a standard siRNA against *Mitf* (*siMitf* [40]) and a modified *Mitf* siRNA with an identical sequence but synthesized to include three 2′-*O*-methyl groups along each strand of siRNA (*siMitf-OM*). In line with MITF repression of ISG expression, knockdown of *Mitf* with either *siMitf* or *siMitf-OM* leads to robust up-regulation of the ISGs *Ifih1*, *Ifit3*, *Irf7*, *Isg15*, and *Stat1* within 48 hours of transfection in comparison to a scrambled siRNA control (*siMitf-scram*; **Fig 4A**). These data support the premise that the IFN signature observed in *Mitf*^*mi-vga9/+*^ mice may be due to MITF-mediated gene regulation of ISGs.

Among the genes differentially expressed in *Mitf*^*mi-vga9/+*^ McSCs, we were particularly interested in IFN regulatory factor 4, *Irf4*. In human melanocytes, MITF binds and activates *Irf4* via an intronic enhancer [42], and in turn, IRF4 can transcriptionally regulate IFN signaling by repressing the master regulatory factor for IFN gene expression, *Irf7* [43,44]. A genome-wide association study of Latin Americans also identified *Irf4* as a locus associated with hair graying [45]. Because *Irf4* and *Irf7* exhibit a reciprocal relationship in *Mitf*^*mi-vga9/+*^ McSCs (*Irf4* is down and *Irf7* is up; **S2 Data**), we asked whether *Irf4* may be indirectly responsible for the up-regulation of ISGs in *Mitf*^*mi-vga9/+*^ melanocytes. However, within the 48-hour time frame that is sufficient for *Mitf* knockdown to result in ISG up-regulation, we did not observe consistent down-regulation of *Irf4* between the two *Mitf* siRNAs (**Fig 4A**). As a control, we further confirmed that cells being treated with either *siMitf* or *siMitf-OM* results in the predictable down-regulation of the melanosomal gene *Pmel17*, which MITF is known to transcriptionally activate (**Fig 4A**; [46]). Based on these observations, we anticipated that the up-regulation of ISGs observed with *Mitf* knockdown cannot be IRF4 dependent. Indeed, using two unique *Irf4* siRNAs (*siIrf4_a* and *siIrf4_b*), *Irf4* gene expression can be significantly reduced in comparison to a negative control siRNA (*siNC1*). However, when considering the results of both *Irf4* siRNAs, *Irf4* knockdown does not lead to the consistent up-regulation or *Irf7* or any of the other ISGs analyzed (**Fig 4B**). Altogether, these results support a novel role for MITF in suppressing the basal IFN signature in melanocytes in vitro and indicate that MITF does not mediate this response through *Irf4*.

An alternate mechanism by which MITF may participate in regulation of innate immune genes is through a direct interaction with their *cis*-regulatory regions, as is the case with many pigmentation genes [46–49]. To investigate this, we screened a previously published MITF chromation immunoprecipitation sequencing (ChIP-seq) dataset for binding of MITF to regulatory regions of innate immune genes [41]. The genomic coordinates of the MITF ChIP-seq peaks found in human primary melanocytes and COLO829 melanoma cells, reported in Webster et al. 2014 [41], were converted from genome build GRCh37/hg19 to NCBI37/mm9 (Galaxy Liftover), and putative target genes were identified using GREAT, version 3.0.0 [50]. For the set of genes that exhibit MITF ChIP-seq peaks within 5 kb of their transcription start site in either direction (6,771), we found 108 genes that are also differentially expressed by greater than 1.5-fold in *Mitf*^*mi-vga9/+*^ McSCs (**Fig 4C**, **S4 Data**). This overlap is under enriched by 1.22-fold compared to expectations (hypergeometric test; *p*-value = 0.0065), suggesting that the majority of gene expression changes observed in *Mitf*^*mi-vga9/+*^ McSCs are indirect. Thus, we further intersected the 108 DEGs with two additional gene lists—one comprising genes reported to be involved in pigmentation (curated from Online Mendelian Inheritiance in Man, Mouse Genome Informatics, and Gene Set Enrichment Analysis) and the second comprising the 55 ISGs identified above in Fig 3A. This comparison validates that, as expected, some DEGs in *Mitf*^*mi-vga9/+*^ McSCs represent known MITF targets involved in pigmentation but also reveals a number of innate immune genes that may also be under MITF transcriptional control (**Fig 4C**). Of the nine innate immune DEGs, seven exhibit an MITF ChIP-seq peak that spans their transcription start site and is congruent with a role for MITF in the direct regulation of these genes at their promotor (**Table 1**). In order to confirm that MITF binds to these loci in mouse melanocytes, we assessed MITF occupancy using chromatin immunoprecipitation quantitative polymerase chain reaction (ChIP-qPCR) for three of the seven ISGs in melan-a cells. Significant chromatin immunoprecipitation (ChIP) enrichment of MITF was observed at the promoter regions of all three of the ISGs predicted to be MITF targets (*Ifih1*, *Ifit3*, and *Stat1*) as well as at the promoter region of a known MITF target gene, *Dct* (**Fig 4D**). No MITF enrichment was observed for the negative control region, a sequence within the centromere. These observations support a direct regulatory role for MITF in the repression of at least some ISGs within melanocytes.

**Table 1:** Relationship of MITF ChIP-seq peaks to the transcription start site (TSS) of innate immune genes.

gene symbol	gene TSS coordinates (mm9)	ChIP-seq peak coordinates (mm9)*	relationship to TSS	RefSeq ID
B2m	chr2: 121973422	chr2: 121972955–121973785	overlapping	NM_009735
Ddx60	chr8: 64406885	chr8: 64406846–64407510	overlapping	NM_001081215
H2-T23	chr17: 36169646	chr17: 36169193–36169773	overlapping	NM_010398
Ifih1	chr2: 62484312	chr2: 62483483–62484952	overlapping	NM_027835
Ifit3	chr19: 34658018	chr19: 34657775–34658283	overlapping	NM_010501
Isg15	chr4: 155574927	chr4: 155576305–155577331	flanking	NM_015783
Lgals9	chr11: 78798426	chr11: 78796426–78797244	flanking	NM_010708
Stat1	chr1: 52176281	chr1: 52175488–52176824	overlapping	NM_009283
Tap1	chr17: 34324500	chr17: 34324057–34325430	overlapping	NM_013683

*ChIP-seq peak coordinates reported by Webster et al. 2014 and converted from genomic build GRCh37/hg19 to NCBI37/mm9 using Galaxy Liftover function.

Abbreviations: ChIP-seq, chromatin immunoprecipitation sequencing; MITF, melanogenesis associated transcription factor; mm9, mus musculus 9 genome build; RefSeq, Reference Sequence database; TSS, transcription start site.

### Tg(Dct-Sox10)/0; *Mitf*^*mi-vga9/+*^ mice exhibit up-regulated ISG expression but no change in the density of immune cells within the skin

**Fig 5:**
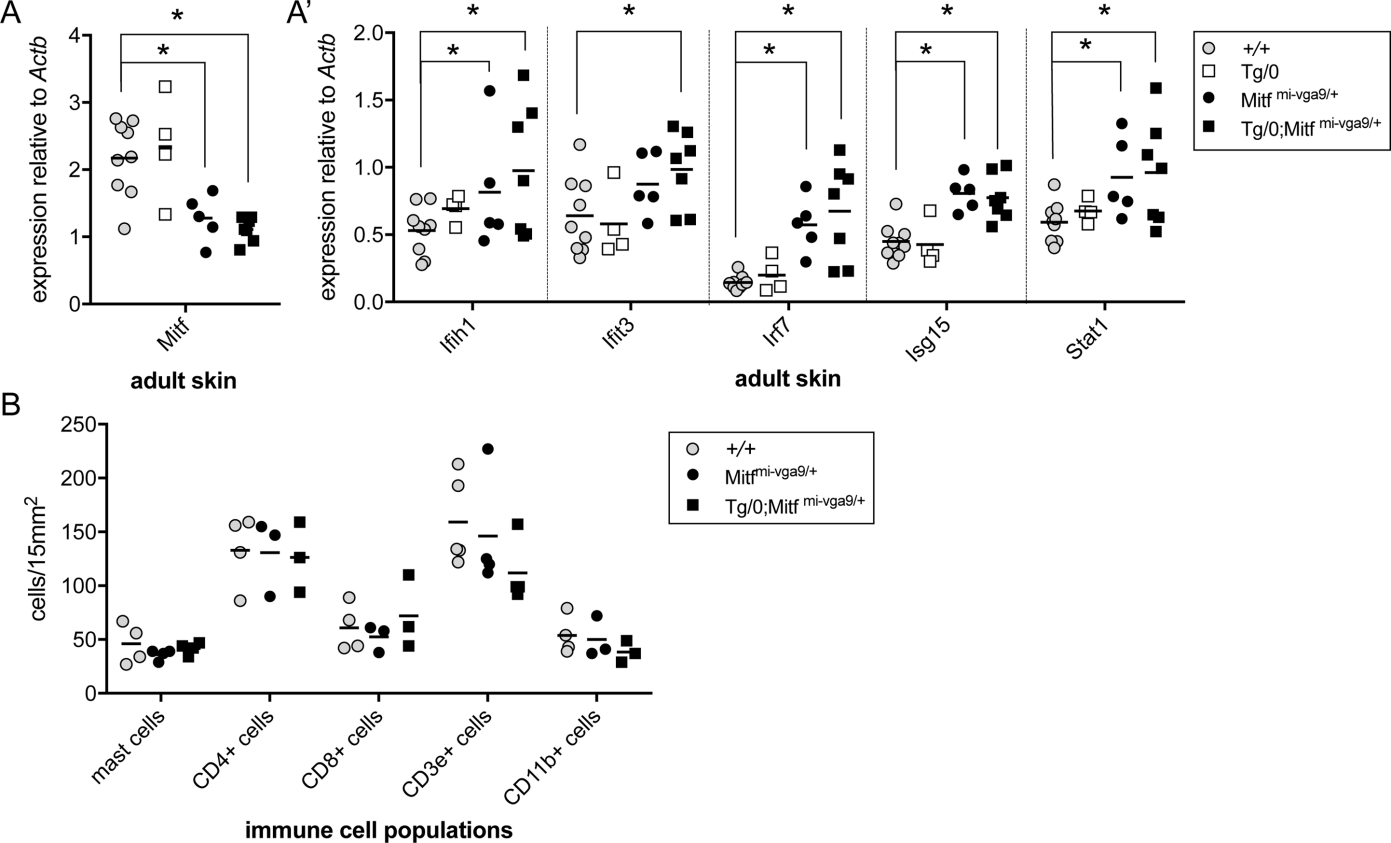
*Mitf*^*mi-vga9/+*^ and Tg(Dct-Sox10)/0; *Mitf*^*mi-vga9/+*^ animals exhibit elevated ISG expression but no change in the density of immune populations within the skin. **(A, A′)** qRT-PCR analysis of *Mitf* and ISG expression in skin isolated from adult wild-type, Tg(Dct-Sox10)/0, *Mitf*^*mi-vga9/+*^, and Tg(Dct-Sox10)/0; *Mitf*^*mi-vga9/+*^ littermates. Each point on the graph represents the expression value from skin of an individual animal. The horizontal bars represent the mean, and the asterisks indicate gene expression changes with a *q*-value of <0.05 using the two-stage linear step-up procedure of Benjamini, Krieger, and Yekutieli, with *Q* = 5%. **(B)** Number of immune-related cells per a 15 mm^2^ area of skin from the same animals as described in (A) harvested at mid-anagen. Mast cells were detected using toluidine blue staining, and other immune cells were detected by immunofluorescence for the indicated markers. Representative images of histological sections used for cell counts are provided in **S2 Fig**. Each point on the graph represents cell counts from an individual animal, and the horizontal bar represents the mean. *t* tests confirmed no statistically significant differences in the density of immune cells when comparing wild-type animals to *Mitf*^*mi-vga9/+*^ or Tg(Dct-Sox10)/0; *Mitf*^*mi-vga9/+*^ animals. The raw data used to generate the graphs in (A), (A′), and (B) are available in **S1 Data**. *Actb*, actin beta; CD, cluster of differentiation; ISG, IFN-stimulated gene; *Mitf*, melanogenesis associated transcription factor; qRT-PCR, quantitative reverse transcription polymerase chain reaction.

While it is interesting that *Mitf*^*mi-vga9/+*^ mice exhibit an IFN signature and that MITF transcriptionally inhibits some of these genes, the real impetus of this study stemmed from previous observations that *Mitf*^*mi-vga9/+*^ causes increased McSC differentiation and hair graying in combination with the transgene Tg(Dct-Sox10) [17]. In order to investigate whether aberrant innate immune signaling might explain hair graying in Tg(Dct-Sox10)/0; *Mitf*^*mi-vga9/+*^ animals, we first evaluated the expression of *Mitf* and ISGs in the skin of adult littermates generated by mating *Mitf*^*mi-vga9/+*^ and Tg(Dct-Sox10)/0 animals. Interestingly, qRT-PCR analysis reveals that skins from Tg(Dct-Sox10)/0 mice exhibit *Mitf* levels similar to that of wild-type, and skins from Tg(Dct-Sox10)/0; *Mitf*^*mi-vga9/+*^mice exhibit reduced levels of *Mitf* that are indistinguishable from *Mitf*^*mi-vga9/+*^ mice (**Fig 5A**). This is in contrast to the expectation that the Tg(Dct-Sox10) transgene would increase *Mitf* expression due to the ability of SOX10 to up-regulate *Mitf* transcriptionally [51]. However, in correlation with these low *Mitf* expression levels, skins from both *Mitf*^*mi-vga9/+*^ and Tg(Dct-Sox10)/0; *Mitf*^*mi-vga9/+*^ mice show a significant up-regulation of the ISGs *Ifih1*, *Irf7*, *Isg15*, and *Stat1*, with Tg(Dct-Sox10)/0; *Mitf*^*mi-vga9/+*^ skins showing the additional up-regulation of *Ifit3* (**Fig 5A′**). Because *Mitf*^*mi-vga9/+*^ mice do not exhibit hair graying themselves [17], these observations suggest that it may be the unique combination of melanocyte dysregulation via Tg(Dct-Sox10)/0 and innate immune dysregulation via *Mitf*^*mi-vga9/+*^ that leads to the exacerbated hair graying observed in Tg(Dct-Sox10)/0; *Mitf*^*mi-vga9/+*^ and Tg(Dct-Sox10)/ Tg(Dct-Sox10); *Mitf*^*mi-vga9/+*^mice.

An IFN signature is commonly detected in affected tissues of patients afflicted with autoimmune disease. Using the same mice as in Fig 5A, we assessed whether changes in resident and infiltrating immune cell populations could provide an etiology for hair graying within Tg(Dct-Sox10)/0; *Mitf*^*mi-vga9/+*^ mice. On cryosections of skin, we quantified mast cells (toluidine blue histologic stain), T cells (CD4+, CD8+, CD3ɛ+), macrophages, and dendritic cells (CD11b). Previous studies report a partial depletion of skin mast cells in *Mitf*^*mi-vga9*^ homozygotes [52]; however, a similar reduction is not observed in *Mitf*^*mi-vga9/+*^ or Tg(Dct-Sox10)/0; *Mitf*^*mi-vga9/+*^ animals. Moreover, immunolabeling for CD3ɛ, CD4, CD8, and CD11b indicates no overt change in the density of these immune cell populations (**Fig 5B, S2 Fig**). Thus, the increased hair graying associated with *Mitf*^*mi-vga9/+*^ is likely not the result of immune cell-mediated destruction of melanocytes.

### Reduction of *Ifih1* is not sufficient to rescue *Mitf*^*mi-vga9*^-mediated hair graying

Recent genome-wide association studies have led to the identification of function-reducing mutations in interferon induced with helicase C domain 1 (*Ifih1*) as protective against vitiligo in humans [53]. *Ifih1* is one of the direct transcriptional targets of MITF identified above (**Fig 4**) and encodes for the protein MDA5. MDA5 sits at the top of the type I innate immune signaling cascade and functions as a cytoplasmic PRR that responds to pathogen-associated molecular patterns (PAMPs) like viral RNA [54]. Gain-of-function mutations of *Ifih1* in humans or overexpression of *Ifih1* in mice is associated with a sustained IFN gene signature and makes it a reasonable candidate for mediating a similar effect downstream of *Mitf*^*mi-vga9/+*^ [55,56]. Thus, we were interested to evaluate whether *Ifih1* haploinsufficiency would be sufficient to reduce the hair graying caused by *Mitf*^*mi-vga9*^ in the context of Tg(Dct-Sox10).

**Fig 6:**
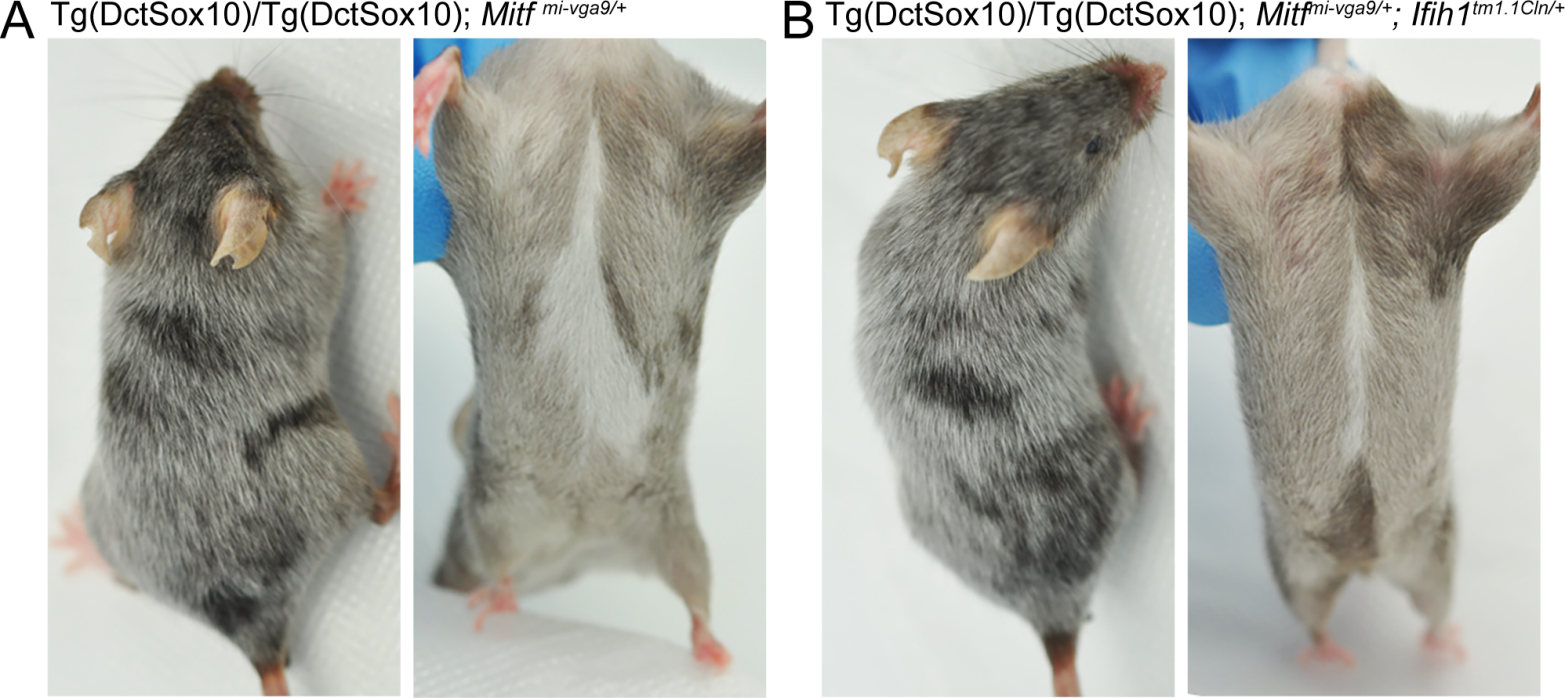
*Ifih1*^*tm1*.*1Cln/+*^ does not affect hair graying in Tg(Dct-Sox10)/Tg(Dct-Sox10); *Mitf*^*mi-vga9/+*^ mice. **(A, B)** Images of Tg(Dct-Sox10)/Tg(Dct-Sox10); *Mitf*^*mi-vga9/+*^ and Tg(Dct-Sox10)/Tg(Dct-Sox10); *Mitf*^*mi-vga9/+*^; *Ifih* ^*tm1*.*1Cln/+*^ littermates imaged at postnatal day 95. **(A)** Tg(Dct-Sox10)/Tg(Dct-Sox10); *Mitf*^*mi-vga9/+*^ animals exhibit a white belly spot at birth and premature hair graying as they age. **(B)** Tg(Dct-Sox10)/Tg(Dct-Sox10); *Mitf*^*mi-vga9/+*^; *Ifih* ^*tm1*.*1Cln/+*^ animals also exhibit hair graying and a belly spot that is slightly reduced in size. Images shown here are representative of five biological replicates for each genotype, with additional images provided in **S3 Fig**. *Ifih1*; interferon induced with helicase C domain 1; *Mitf*, melanogenesis associated transcription factor.

Hair graying associated with Tg(Dct-Sox10) is most readily visible in mice that are homozygous for the transgene, with Tg(Dct-Sox10)/Tg(Dct-Sox10); *Mitf*^*mi-vga9/+*^ animals exhibiting robust graying at 110 days (**Fig 1**). Comparing Tg(Dct-Sox10)/Tg(Dct-Sox10); *Mitf*^*mi-vga9/+*^ mice to their littermates, we find that additional heterozygosity for *Ifih1* (*Ifih* ^*tm1*.*1Cln/+*^) does not reverse hair graying to the levels observed in Tg(Dct-Sox10)/Tg(Dct-Sox10) mice (**Fig 6, S3 Fig**). At most, and in a qualitative sense, *Ifih* ^*tm1*.*1Cln/+*^ appears to produce a small reduction in the existing congenital white belly spot. These observations suggest that while MITF may repress *Ifih1* in melanocytes in vitro, reduction of *Ifih1* in vivo is insufficient to reverse hair graying associated with *Mitf*^*mi-vga9/+*^. This may indicate that the influence of MITF in innate immune regulation extends beyond the regulation of an individual gene, which is consistent with the fact that *Ifih1* is not the only innate immune target gene directly downstream of MITF (as shown in **Fig 4**).

### Systemic poly(I:C) administration exacerbates hair graying in genetically susceptible mice

Up to this point, it is still unclear whether the innate immune dysregulation associated with *Mitf*^*mi-vga9*^ contributes to elevated hair graying in Tg(Dct-Sox10) mice or whether these two phenomena are merely correlated. To investigate if the activation of innate immune signaling is sufficient to mediate changes in McSC maintenance and hair graying independent of *Mitf*, we asked if hair graying can be induced in mice exposed systemically to the viral mimic poly(I:C). Poly(I:C) administered in vivo by intraperitoneal injection induces type I IFN production, which is detectable in the blood serum and lymphoid system [37,57]. This in turn initiates the antiviral response via up-regulation of ISGs to limit viral replication [58]. To mimic the combination of *Mitf*^*mi-vga9*^ and Tg(DctSox10) that leads to exacerbated hair graying, we examined Poly(I:C)-mediated innate immune activation on the Tg(Dct-Sox10) background and compared it to wild-type.

**Fig 7:**
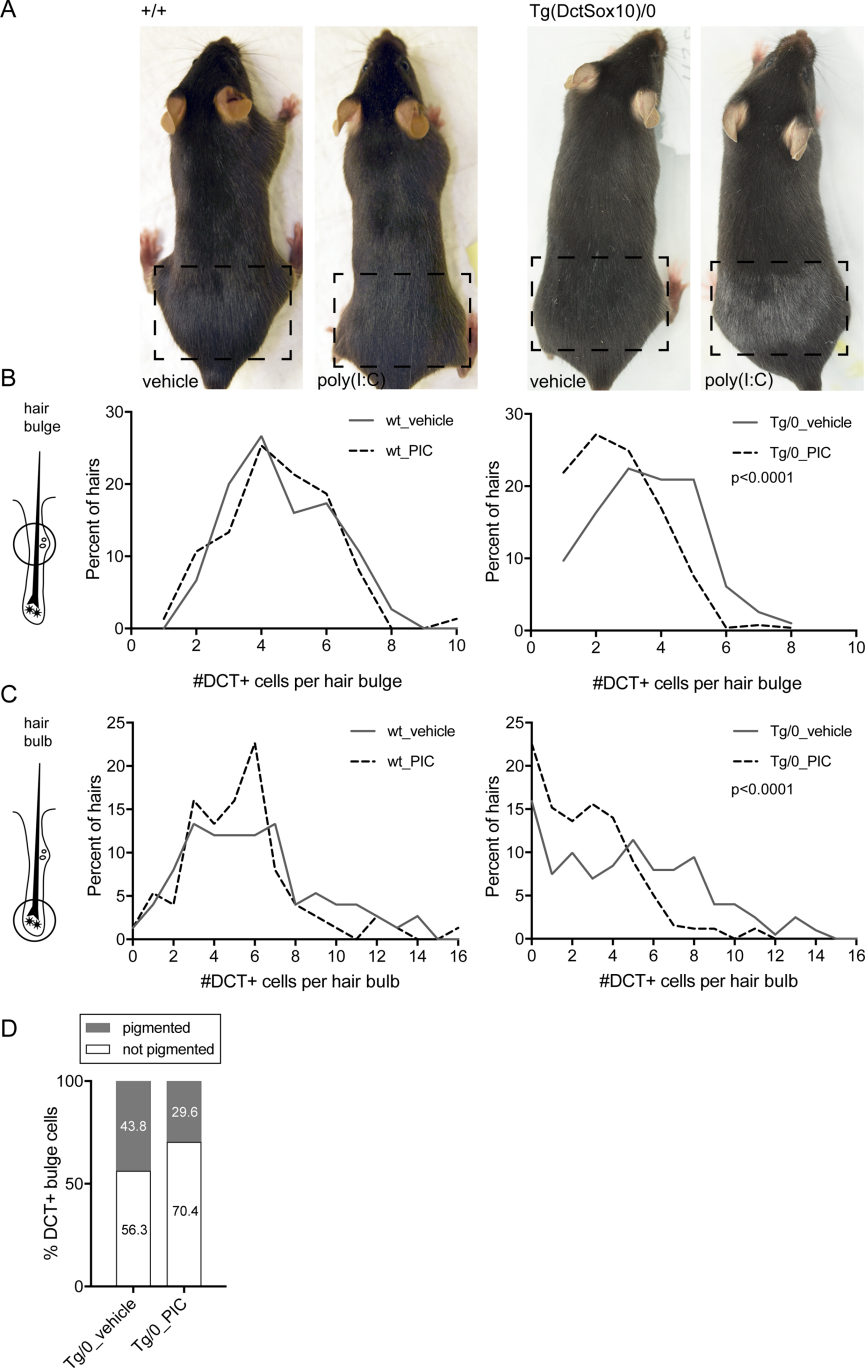
Treatment with the viral mimic poly(I:C) worsens hair graying in Tg(Dct-Sox10)/0 susceptible mice. **(A)** Wild-type (left) and Tg(Dct-Sox10)/0 (right) mice were plucked along their lower back (the plucked region is indicated by the dashed boxes) to synchronize and initiate the hair cycle in this area. Starting on the fourth day after plucking, mice received an intraperitoneal injection on three consecutive days of 100 μL of physiological water alone (vehicle) or physiological water containing 100 μg of poly(I:C). Hair regrowth was allowed to proceed normally and the mice were imaged within the same hair cycle, approximately 21 days after the initial plucking. Images shown here are representative of 3–5 animals for each genotype and treatment. **(B, C)** Histograms of the percent of hairs exhibiting DCT+ McSCs in the hair bulge (B) or DCT+ melanocytes in the hair bulb (C) of mice treated similarly to those described in (A). For this experiment, skins were harvested on the seventh day after plucking, sectioned, and immunolabeled for DCT. For the wild-type mice, each histogram represents 75 hairs evaluated across 3 animals for each anatomic location. For the Tg(Dct-Sox10)/0 mice, each histogram represents >190 hairs evaluated across 6 animals for each anatomic location. Histogram distributions were assessed for significance using the Kolmogorov-Smirnov test. **(D)** Quantification of pigmented and not pigmented DCT+ McSCs within the hair bulge of mice treated and prepared similarly to those described in (B). For the Tg(Dct-Sox10)/0 mice injected with the vehicle control, 256 McSCs were assessed across 3 animals. For the Tg(Dct-Sox10)/0 mice injected with poly(I:C), 260 McSCs were assessed across 5 animals. The difference in the proportion of pigmented and not pigmented DCT+ McSCs between treatments was tested for significance using the Fisher’s exact test (*p* = 0.001). The raw data used to generate the graphs in (B), (C), and (D) are available in **S1 Data**. DCT, dopachrome tautomerase; McSC, melanocyte stem cell; poly(I:C); polyinosinic:polycytidylic acid.

To assess the effects of poly(I:C) on hair pigmentation, hairs along the lower back of each mouse were plucked to synchronize and activate the hair cycle, and the mice were injected with poly(I:C) for 3 days sequentially, beginning at 4 days post plucking. The presence of nonpigmented, gray hairs is detected by visual inspection once the hairs have emerged from the skin. Notably, treatment with this viral mimic is sufficient to increase the amount of gray hairs present in the plucked area in Tg(Dct-Sox10)/0 mice but not adequate to induce hair graying on the wild-type background (**Fig 7A**). By immunolabeling for the melanocyte marker DCT on skin sections from these mice, we demonstrate that this hair graying phenotype is the result of the quantitative reduction of McSCs (DCT+ cells in the hair bulge) and melanocytes (Dct+ cells in the hair bulb; **Fig 7B and 7C**). This same cell loss is not observed in wild-type animals that are treated with poly(I:C). Taken together, these results suggest that proper regulation of innate immune signaling during active hair growth is essential to both McSCs and differentiating melanocytes and that this effect is unmasked by the Tg(Dct-Sox10) genetic background. Akin to Tg(Dct-Sox10)/0; *Mitf*^*mi-vga9/+*^ animals, treating Tg(Dct-Sox10)/0 mice with poly(I:C) leads to increased hair graying.

In order to investigate the cellular mechanism responsible for the loss of McSCs and melanocytes in Tg(Dct-Sox10)/0 mice treated with poly(I:C), we performed further histological analysis to assess for T-cell infiltration and apoptosis. Using immunolabeling and the pan T-cell marker CD3ε, we found no qualitative difference in T-cell localization or density within the skins of Tg(Dct-Sox10)/0 mice injected with poly(I:C) or the vehicle control (**S4 Fig**). Double labeling for DCT and the apoptotic marker, cleaved caspase 3 (CC3), also revealed no apparent CC3+ cells within the hair follicles where McSCs or melanocytes reside (**S4 Fig**). Interestingly, however, closer examination of the differentiation state of the McSCs in Tg(Dct-Sox10)/0 mice treated with poly(I:C) revealed a significant reduction in the percentage of ectopically pigmented McSCs within the hair. Previously, we established that about half of the McSCs in Tg(Dct-Sox10)/0 mice exhibit a differentiated phenotype in comparison to wild-type [17]. However, the proportion of ectopically pigmented McSCs in Tg(Dct-Sox10)/0 mice treated with poly(I:C) is significantly lower than in Tg(Dct-Sox10)/0 mice given the vehicle control (**Fig 7D**). We surmise that the drop in overall McSC numbers observed in poly(I:C)-treated Tg(Dct-Sox10)/0 mice (shown in Fig 7B) is due to the specific loss of McSCs that have inappropriately differentiated, and that the loss of the these McSCs either occurred at an earlier time point or via a mechanism that does not involve T-cell infiltration or CC3-associated apoptosis. Interestingly, hair graying in Tg(Dct-Sox10)/0; *Mitf*^*mi-vga9/+*^ mice is more progressive than the acute graying observed in Tg(Dct-Sox10)/0 mice treated with poly(I:C) [17], and hair graying in Tg(Dct-Sox10)/0; *Mitf*^*mi-vga9/+*^ mice is preceded by excessive McSC differentiation, a phenotype that we do not observe in the Tg(Dct-Sox10)/0 mice treated with poly(I:C). In the future, it will be important to evaluate whether lower dosages of poly(I:C) can drive McSC differentiation or whether these two hair graying models represent distinct cellular pathologies.

## Discussion

By assessing McSCs and melanoblasts in vivo and melanocytes in vitro, we identify a novel role for MITF in the transcriptional repression of target genes involved in type I innate immune signaling. Accordingly, we demonstrate that haploinsufficiency for *Mitf* incites a heightened and sustained IFN gene signature in McSCs, melanoblasts, and whole skin, and that *Mitf*^*mi-vga9/+*^ melanoblasts have an enhanced ability to respond to viral mimic. We show through siRNA knockdown and ChIP analysis in vitro that MITF transcriptionally represses several ISGs and that for some of these ISGs, this response may be through the direct binding of MITF to their gene promoter. Furthermore, we establish that postnatal melanocytes and McSCs are negatively affected by systemic activation of innate immune signaling in vivo and that this contributes to the pathogenesis of hair graying when considered in the context of a genetic background that is already at risk for this phenotype.

The participation of MITF in the regulation of innate immune target genes within the melanocyte lineage is unanticipated but is reported within other genomic datasets. For instance, RNA-seq analysis of human melanoma COLO829 cells transfected with *MITF* short hairpin RNA (shRNA) demonstrates an up-regulation of over 17 of the same 55 innate immune DEGs identified in *Mitf*^*mi-vga9/+*^ McSCs, including *Ifih1*, *Ifit3*, *Irf7*, *Parp9*, and *Stat1* (GSE50686 [41]). Differential regulation of 15 of the 55 *Mitf*^*mi-vga9/+*^ innate immune DEGs is also observed after transient knockdown of *MITF* in the immortalized, human melanocytes Hermes 3A, although the direction of expression varies in comparison to our observations [59]. Interestingly, animals that are homozygous for a loss-of-function mutation in the melanocortin 1 receptor gene (*Mc1r*^*e/e*^) also exhibit differential expression of ISGs in skin and spleen [60]. MC1R and MITF exist in a positive feedback regulatory loop, in which MC1R activation leads to cAMP/CREB-dependent up-regulation of *Mitf* [61], and MITF acts as a transcriptional activator of *Mc1R* gene expression [47,62]. These observations further highlight a link between key melanocyte proteins and the expression of genes involved in the innate immune response.

The involvement of MITF in ISG regulation and hair graying is particularly relevant in light of the increasing number of studies indicating an innate immune component to vitiligo [63]. In humans, association studies identified *IFIH1* as well as the innate immune genes, NLR family pyrin domain containing 1 (*NLRP1*) and toll like receptor adaptor molecule 1 (*TICAM1*), as susceptibility loci associated with generalized vitiligo [53,64–66]. *NLRP1* and *TICAM1* encode the proteins for a PAMP receptor involved in microbial sensing and an adapter molecule involved in innate immune Toll-like receptor signaling, respectively. Infiltrations of active natural killer cells and inflammatory dendritic cells, both innate immune effector cells, are also observed in the lesional skin of vitiligo patients [67,68]. In the Smyth line chickens, an avian model of spontaneous vitiligo, global gene expression changes associated with the innate immune pathway are observed during both the onset and progression of vitiliginous disease [69]. Additionally, vaccination of these birds with live turkey herpesvirus greatly increases the incidence of hypopigmentation in this susceptible avian line [70,71]. Further investigation into the molecular mechanisms that drive the hair graying phenotypes associated with McSC and melanocyte loss in Tg(Dct-Sox10)/0; *Mitf*^*mi-vga9/+*^ mice or Tg(Dct-Sox10)/0 mice treated with poly(I:C) may provide new insight into the role of innate immune regulation in hypopigmentary disease.

We do not yet understand why only the Tg(Dct-Sox10) mice are sensitive to poly(I:C). Some have speculated that melanocytes produce byproducts that act as endogenous damage-associated molecular patterns (DAMPs) in response to cellular stress [72]. DAMPs, like PAMPs, have the capacity to initiate PRR activation but do so in the absence of virus in a process known as sterile inflammation. Within Tg(Dct-Sox10) mice, overexpression of *Sox10* drives the production of aberrant pigmentation with the McSCs [17], and we can now use the Tg(Dct-Sox10) line as a model to ask whether this process leads to the triggering of damage-associated innate immune effectors.

Few reports describe loss of hair pigmentation or vitiligo-like symptoms in mammalian model systems or human patients after exposure to virus, viral mimic, or elevated type I IFN. In a set of case reports of IFN-α-induced vitiligo after treatment of hepatitis C, it is postulated that the reason hair graying is infrequently observed in hepatitis C patients is because IFN treatment is “only sufficient to unmask vitiligo in susceptible individuals” [73]. This idea is encouraged by our observations that neither *Mitf*^*mi-vga9/+*^ nor poly(I:C) treatment on its own is sufficient to induce changes in postnatal pigmentation in mice, but both can modify hair graying when combined with the susceptible Tg(Dct-Sox10) background. Other examples demonstrate that even with an elevated basal IFN signature, additional environmental or genetic factors are often required to unmask autoimmune disorders, suggesting that this phenomenon is not limited to melanocyte pathologies. For instance, mice expressing multiple copies of an *Ifih1* transgene spontaneously generate type I IFN and exhibit no overt phenotype, but increase disease severity when crossed with a lupus-prone strain [55]. Genetic variations in *Ifih1* in humans are also associated with increased type I IFN signaling and an increased risk for the development of a number of autoimmune disorders, including Aicardi-Goutieres syndrome, systemic lupus erythematosus, and type I diabetes [56,74,75].

Finally, it is important to mention that while this study has focused on assessing the potential role of MITF in the regulation of ISGs autonomous to the melanocyte, these data do not exclude the possibility that a nonautonomous source of IFN also contributes to the ISG signature observed in the melanoblasts and McSCs isolated from *Mitf*^*mi-vga9/+*^ animals. For instance, in addition to melanocytes, MITF is also indispensable for proper mast cell development. Beyond their well-known role in the allergic response, mast cells are also recognized as effectors and modulators of the innate immune response [76,77]. While we did not observe a change in the number of mast cells in *Mitf*^*mi-vga9/+*^ mice (**Fig 5B**), other studies have reported that animals that are homozygous for *Mitf*^*mi-vga9*^ show significantly reduced mast cell numbers, defects in mast cell differentiation and proliferation, and a reduced ability to resist bacterial infection [52,78,79]. Conditional rescue of *Mitf* expression within the melanocyte lineage of *Mitf*^*mi-vga9/+*^ mice would help determine whether nonautonomous mechanisms contribute to ISG expression in melanocytic cells and would further refine our understanding of the interplay between melanocyte biology and innate immune signaling.

In summary, we describe a negative regulatory role for MITF in the expression of innate immune genes in melanocytes in vitro and establish the *Mitf*^*mi-vga9/+*^ mouse line as one in which the McSCs, melanoblasts, and whole skin exhibit a chronically elevated IFN signature. This finding describes a new mechanism by which melanocytes can intrinsically regulate their innate immune response, the implications of which are relevant to both melanocyte physiology and disease. Our data also highlight poly(I:C)-treated, Tg(Dct-Sox10) mice as one of very few examples of an in vivo, innate immune-related, hair graying mouse model. The fact that genetically distinct mice respond differently to poly(I:C) is noteworthy and may help to explain anecdotal stories in humans of spontaneous hair graying after viral infection.

## Materials and methods

### Ethics statement

All research completed in the manuscript was performed under the guidance of the UAB IACUC or NIH IACUC and is associated with the following animal protocols: UAB 20382 (to MLH); NCI-Frederick 17–031 (to GM); NHGRI G-94-7 (to WJP).

### Animals, mouse crosses, genotyping

*Mitf*^*mi-vga9*^ mice on a mixed C57BL/6 × C3H genetic background were received as a gift (H. Arnheiter [80]) and rederived on C57BL/6J. *Mitf*^*mi-vga9*^ mice were maintained by intercross and backcross to C57BL/6J. To generate the *Mitf*^*mi-vga9/+*^ mice for RNA-seq analysis (**Fig 3**, **S2 Data**), *Mitf*^*mi-vga9*^ homozygotes were backcrossed to C57BL/6J mice. To generate the wild-type mice for RNA-seq analysis, C57BL/6J mice were intercrossed. The wild-type and *Mitf*^*mi-vga9/+*^ mice used for the qRT-PCR of skin in **S1 Fig** were generated as part of an unrelated study and are also homozygous for the Dct-rtTA transgene [81]. The Dct-rtTA transgene is used for doxycycline-induced expression of tetracycline responsive elements in the presence of doxycycline. These animals were age-matched control animals and as part of this unrelated experiment were not treated with doxycycline. The +/+; rtTA/rtTA animals were generated by mating +/+; rtTA/rtTA and +/+; rtTA/rtTA animals. The *Mitf*^*mi-vga9/+*^; rtTA/rtTA animals were generated by using *Mitf*^*mi-vga9/ mi-vga9*^; rtTA/rtTA animals that were also heterozygous for a tetracycline inducible *Mitf* transgene and mating them with +/+; rtTA/rtTA animals. To generate the *Mitf*^*mi-vga9/+*^ and wild-type animals used for primary melanoblast isolation (**Fig 3**), *Mitf*^*mi-vga9/+*^ mice were backcrossed to C57BL/6J mice and the littermates were used for analysis.

The Tg(Dct-Sox10) transgenic line was generated on the FVB/N background and maintained through a combination of backcrossing to C57BL/6J and by intercross (Tg(Dct-Sox10)CF1-10Pav [82]). To generate the double and single mutants used for the analysis of gene expression in skin (**Fig 5**), *Mitf*^*mi-vga9/+*^ mice were crossed to Tg(Dct-Sox10)/0 mice and the littermates used for analysis. To generate the Tg(Dct-Sox10)/0 for treatment with poly(I:C) (**Fig 7**), Tg(Dct-Sox10) homozygotes were backcrossed to C57BL/6J mice.

*Ifih* ^*tm1*.*1Cln*^ homozygote mice were obtained from JAX (015812, [37]) and crossed to Tg(Dct-Sox10)/0 mice. The Tg(Dct-Sox10)/0; *Ifih* ^*tm1*.*1Cln*/+^ progeny from this cross were mated further with Tg(Dct-Sox10)/Tg(Dct-Sox10); *Mitf*^*mi-vga9/+*^ mice to assess *Ifih* ^*tm1*.*1Cln*^ rescue (**Fig 6**).

All mice were genotyped using DNA isolated from tail tips and PCR analysis. Primers and cycling conditions for the *Mitf*^*mi-vga9*^ and Tg(Dct-Sox10) alleles were described previously [17]. The *Ifih* ^*tm1*.*1Cln*^ allele was amplified under standard PCR conditions (30 cycles of 45 seconds at 94°C, 45 seconds at 65°C, and 60 seconds at 72°C), using the genotyping primers described at JAX: Ifih1_common_reverse, CTTCTGTTCTTCCTGGGAGACC; Ifih1_wildtype_forward, GGGACTATTGACAGTCGAAGAC; and Ifih1_mutant_forward, CCCCGGTCAAAGCTGAATAAAT.

### FACS isolation of McSCs and melanoblasts

Single cell dermal isolations were generated from P1.5 or 8-week-old mice and sorted by FACS to obtain melanoblasts or McSCs, respectively. Cells of the melanocyte lineage were identified by their surface expression of the receptor KIT. Both melanoblasts and McSCs reside in the hair follicle at these respective time points and exhibit KIT+ immunolabeling (**Figs 2 and 3**). This isolation method was adapted from previously published protocols [83][84]. In brief, trunk skin was incubated in 0.25% Trypsin-EDTA to separate the dermis from the epidermis. The dermis was further dissociated by enzymatic treatment with 0.3 mg/mL Liberase TL (Roche) and then physically disrupted by passing the dermal cell solution forcefully through a syringe-type, 70 uM Filcon filter (BD Biosciences). Dermal cells were labeled with the cell surface markers, KIT (CD117, BD Pharmingen) and CD45.2 (BD Pharmingen), and assessed for fluorescence on a FACSAria (Becton Dickinson). Cells of the melanocyte lineage were positively selected for by gating on the KIT+, CD45.2− cell population. Mast cells were negatively selected away by removing any double positive cells (KIT+, CD45.2+) (**Fig 2**). Analysis of the cell populations within FACS gates was performed using FlowJo software.

### RNA isolation and qRT-PCR

Cells and tissues for qRT-PCR analysis were harvested in TRIzol reagent (ThermoFisher) and tissues homogenized using a BeadBlaster (Benchmark). Total RNA from tissue, FACS cells, or tissue culture cells was purified using the Directzol RNA MiniPrep Kit (Zymo) and quantified on a Qubit fluorometer or Biotek spectrophotometer. For qRT-PCR analysis, 1–2 ug of RNA was reverse transcribed using the High Capacity cDNA Reverse Transcription Kit (ABI). qRT-PCR was performed on this cDNA using Taqman gene expression assays and Taqman Fast Universal PCR Master Mix. The gene expression assays used here include: *Actb*, mm00607939_s1; *Ifih1*, mm00459183_m1; *Ifit3*, mm01704846_s1; *Ifna4*, mm00833969_s1; *Ifnb1*, mm00439552_s1; *Irf4*, mm00516431_m1; *Irf7*, mm00516793_g1; *Isg15*, mm01705338_s1; *Mitf*, Mm00434954_m1; *Pmel*, mm00498996_m1; *Stat1*, Mm00439531_m1.

### RNA-seq and differential gene expression analysis

McSCs from approximately ten 8-week-old animals were pooled to generate enough RNA to serve as one biological replicate. RNA quality for RNA-seq was assessed by Bioanalyzer (Agilent), and only samples with RIN scores >7.5 were used. mRNA libraries were constructed from about 200 ng total RNA using the Illumina TruSeq RNA Sample Prep Kits, version 2. The resulting cDNA was fragmented using a Covaris E210. Library amplification was performed using 16 cycles. Unique barcode adapters were applied to each library. Libraries were pooled in equimolar ratio and sequenced together on a HiSeq 2000 with version 3 flow cells and sequencing reagents. At least 54 million 101-base read pairs were generated for each individual library. Data were processed using RTA 1.13.48 and CASAVA 1.8.2.

RNA from each sample was sequenced on two separate lanes, obtaining paired-end reads that were 101 bp in length. RNA-seq reads that passed the Illumina platform quality check were used for downstream analyses. RNA-seq reads were aligned to the mouse mm9 reference genome sequence with the STAR (v. 2.3.0e) software using default parameters. RNA-seq reads that mapped to genomic locations for known rRNAs (obtained from the UCSC genome browser) were filtered using split_bam.py included in the RSeQC package (v. 2.3.7). RNA-seq read counts were calculated with the htseq_count (v. 0.5.3p3) software using annotations for protein coding genes obtained from Ensembl (release 67), thus obtaining counts for RNA-seq reads that mapped unambiguously to Ensembl gene models. These RNA-seq counts were used for differential gene expression analysis performed with DESeq2. For the differential gene expression analysis, a comparison of RNA-seq reads counts between two experimental conditions was done, with each condition containing RNA-seq data collected from three biological replicates. The RNA-seq data produced in this study are available at NCBI GEO under accession GSE102271.

To make the heatmap, a regularized log transformation was applied (rlog) to the DESeq2 results data frame (DEseq2). These values for the genes of interest were loaded into the heatmap R package and scaled and clustered by row to generate the heatmap.

### Cell culture and siRNA transfection

Primary melanoblasts obtained from FACS were grown in RPMI 1640 medium supplemented with 10% fetal bovine serum (not heat inactivated, Gemcell), 200 nM 12-O-tetradecanoylphorbol 13-acetate (TPA, Sigma-Aldrich), 10 ng/mL stem cell factor (Gibco), 0.6 ng/mL basic fibroblast growth factor (Stemgent), 2 mM glutamine, 100 U/mL penicillin, and 100 ug/mL streptomycin. Cells from the immortalized, mouse melanocyte cell line, melan-a (melan-Ink4a-Arf-1 [85]) were grown in RPMI 1640 medium supplemented with 10% fetal bovine serum (Gibco), 200 nM TPA (Sigma-Aldrich), 200 pM cholera toxin (Sigma- Aldrich), 2 mM glutamine, 100 U/mL penicillin, and 100 ug/mL streptomycin. Primary and immortalized cells were incubated at 37°C in humidified air with 10% CO_2_.

siRNA-mediated gene knockdown was performed over 48 hours using Lipofectamine RNAiMAX (Invitrogen) and the following duplex siRNAs (IDT and IDT TriFECTa): *siMitf-scram* (5′-UAUCUUUAACCGUUCGUCCAGUGC-3′, 3′-TTAUAGAAAUUGGCAAGCAGGUCACG-5′); *siMitf* (5′-GGUGAAUCGGAUCAUCAAGCAAGATT-3′, 3′-CCACUUAGCCUAGUAGUUCGUUCU-5′); *siMitf-OM* (same sequence as *siMITF* with 3, 2′-*O*-methyl-modified uridines along each siRNA); *siIrf4_a* (5′-CUUGGAAGACAAGAUUACGAUGUGC-3′, 3′-AUGAACCUUCUGUUCUAAUGCUACACG-5′); *siIrf4_b* (5′-GGGCAUUGUUUAAAGGAAGUUCCG-3′, 3′-AACCCGUAACAAAUUUCCGUUCAAGGC-5′) and *siNC1* (negative control #1, Thermofisher).

### ChIP assay

ChIP assay was performed on chromatin lysates extracted from melan-a cells. Two million cells were cross-linked at room temperature for 10 minutes using 1% formaldehyde (final concentration), and chromatin lysates were prepared according to a previously described protocol [86]. The ChIP reaction was carried out by incubating chromatin lysates with anti-MITF antibody (HPA003259; Sigma-Aldrich, Inc., St. Louis, MO). IgG ChIP was performed with a nonspecific antibody control. DNA coprecipitating with anti-MITF and anti-IgG antibodies was recovered on magnetic beads (Dynabeads, Invitrogen), de–cross-linked, and purified using the *phenol*:*chloroform*:*isoamyl* alcohol DNA extraction method. DNA obtained from ChIP as well as input (lysates with no antibody added) was quantified by RT-PCR using primer sets for promoter regions of Stat1, Ifit3, Ifih1, Dct, and a negative control region. DNA sequences for the primer sets used in the experiment are as follows: *Stat1* (Forward, GACCAAGTGTCCGGGACTAA; Reverse, TGCTTTCTTCCAAGTGTCACAG); *Ifit3* (Forward, CAGTGATGCACATGTTACCAA; Reverse, CCAGCTTTTAATGACTACGTCCA); *Ifih1*(Forward, ACAGGGACCTTGCATACTGG; Reverse, GGCCTGCCTAATGACAGATG); *Dct* (Forward, CCTGACACAAAGCCAGACAC; Reverse, GAAGCCATCATTAAGGGGATT); negative control region (Catalog#: 71018; Active Motif). Quantitative RT-PCR reaction was performed using the SYBR Green dye protocol (Invitrogen) and data were analyzed using the relative C_t_ method (2^(−ΔCт)^), as described in the Applied Biosystems user manual. ChIP enrichment signals are presented as percent input (input normalization). All ChIP data are from two independent biological replicate experiments.

### Poly(I:C) administration

Poly(I:C) (high molecular weight, InvivoGen) for in vitro transfection was first complexed with Lipofectamine 2000 (375 ng poly(I:C)/uL lipofectamine reagent). This complex was added to cell culture growth medium to achieve a final concentration of 250 ng/mL poly(I:C). Primary melanoblasts transfected with this complex were harvested after 9 hours.

Animals treated with poly(I:C) first had a small region of hair removed from their lower back via hand plucking. Four days after plucking, animals were administered an intraperitoneal injection of 100 uL of physiological water with or without 100 μg of poly(I:C). Skin for immunolabeling was harvested from the plucked region of these animals on 7 days post plucking, and animals for live imaging were photographed at approximately 21 days post plucking.

### Histological staining and immunofluorescence

Skin isolated from the lower back of plucked mice was immersed in 2% formaldehyde for 30 minutes on ice. Skins were cryoprotected in 10% sucrose overnight, embedded in NEG-50 (Thermo Scientific), frozen, and sectioned with a cryostat (8–10 μm).

For identifying mast cells, skin sections were stained for 2 minutes in toluidine blue solution (18.7 mM toluidine blue, 166 mM urea, 30% ethanol), washed in water, dehydrated in 50% ethanol, and then mounted with Permount. For immunofluorescence, sections were washed in PBS with 0.1% Tween 20, optionally blocked in 1% BSA, and incubated in primary antibody overnight at 4°C. After washing, the sections were incubated in the appropriate secondary antibody (1:2,000; AlexaFluor, Invitrogen) for 2 hours at room temperature.

Immune cells were detected using primary antibodies against CD3ɛ (1:50, KT3, Thermofisher), CD4 (1:200, GK1.5, Thermofisher), CD8 (1:200, 53–6.7, Thermofisher), and CD11b (1:1,000, M1/70, Abcam). Quantitation of immune cells was performed by taking five images along a length of skin at 7 dpp (about 5.25 mm total imaged length, see representative images in **S2 Fig**) and counting the number of positive cells within the epidermis, dermis, and subcutis (about 15 mm^2^ total area).

Melanocytes and McSCs were detected using a primary antibody against DCT (1:300; TRP2, Santa Cruz Bio). McSCs and melanocytes were defined as those DCT+ cells residing in the hair bulge (near the border between the dermis and the subcutis) and the hair bulb (surrounding the hair matrix), respectively. Quantitation of DCT+ cells per hair was performed on every fourth section of sequentially obtained skin sections. Apoptotic cells were identified using a primary antibody against CC3 (1:100, Cell Signaling).

Bright-field and fluorescence microscopy and imaging were performed either on an Observer.D1 microscope with an Axiocam Hrc camera (Zeiss) or on an EVOS FL Cell Imaging System (Thermofisher). Images were post processed using Adobe Photoshop.

## Supporting information

S1 Data Raw data and statistical analysis used to generate graphs. (XLSX)

S2 Data List of genes that are differentially expressed (>1.5-fold) in wild-type and *Mitf^mi-vga9/+^* McSCs based on RNA-seq analysis. BaseMean represents the average of the normalized count values, dividing by size factors, taken over all six samples within the dataset. Log2(fold change) represents the binary logarithm of wild-type over *Mitf*^*mi-vga9/+*^ gene expression values, and lfcSE represents the log2(fold change) standard error. Stat refers to the Wald statistic used to determine the *p-*value. Both the unadjusted (pvalue) and Benjamini-Hochberg adjusted (padj) *p*-values are provided, with significant genes being those with a padj <0.05. lfcSE, log2(fold change) standard error; McSC, melanocyte stem cell; *Mitf*, melanogenesis associated transcription factor; padj, Benjamini-Hochberg adjusted *p*-value; pvalue, unadjusted *p*-value; RNA-seq, RNA sequencing.
(XLSX)

S3 Data DAVID Functional Annotation Cluster results using a list of genes that are up-regulated (>1.5-fold) in *Mitf^mi-vga9/+^* McSCs (spreadsheets “DAVID on 1.5-fold up genes,” “gene list from cluster1,” “gene list from cluster2”). Sixty-five unique genes from annotation clusters 1 and 2 were combined (spreadsheet “65 genes in cluster 1+2”). These 65 genes were queried at interferome.org for their designation as IFN-regulated genes (“55 IRGs interferome.org”). Fifty-five of the sixty-five genes identified by DAVID exhibit a greater than 2-fold gene expression change in mice or mouse cells in response to IFN-α or IFN-β (spreadsheets “55 IRGs interferome.org data,” “55 IRGs list”). IFN, interferon; McSC, melanocyte stem cell; *Mitf*, melanogenesis associated transcription factor.
(XLSX)

S4 Data List of “MITF direct target” genes. These are genes that are differentially regulated (>1.5-fold) in *Mitf*^*mi-vga9/+*^ McSCs in comparison to wild-type McSCs and exhibit an MITF ChIP-seq peak. MITF ChIP-seq peaks (Webster et al. 2014) were associated with nearby genes using GREAT (peaks that land ± 5 kb from the transcription start site). ChIP-seq, chromatin immunoprecipitation sequencing; GREAT, genomic regions enrichment of annotations tool; McSC, melanocyte stem cell; MITF, melanogenesis associated transcription factor.
(XLSX)

S1 Fig qRT-PCR analysis of *Mitf* and ISG expression (*Ifih1*, *Ifit3*, *Irf7*, *Isg15*, and *Stat1*) in skins of +/+; rtTA/rtTA and *Mitf^mi-vga9/+^*; rtTA/rtTA mice isolated at P32. These mice were housed in the animal facility independent from those where the remainder of this study was performed. Each point on the graph represents the expression value from skin of an individual animal. The horizontal bars represent the mean, and the asterisks indicate gene expression changes with a *q*-value of <0.05 using the two-stage linear step-up procedure of Benjamini, Krieger and Yekutieli, with *Q* = 5%. ISG, interferon stimulated gene; *Mitf*, melanogenesis associated transcription factor; P, postnatal day; qRT-PCR, quantitative reverse transcriptase polymerase chain reaction; rtTA, reverse tetracycline-controlled transactivator.
(TIF)

S2 Fig Histological and immunofluorescent staining of immune cell populations in mid-anagen skin isolated from wild-type (left), *Mitf^mi-vga9/+^* (center), and Tg(Dct-Sox10)/0; *Mitf^mi-vga9/+^* (right) animals. **(A)** Mast cells were detected using toluidine blue and were found dispersed throughout the dermis. **(B–D)** Antibodies to CD3ɛ, CD4, and CD8 were used to identify T cells within the epidermis and the dermis. **(E)** Antibodies against CD11b were used to detect macrophages and Langerhan’s cells and these were distributed within dermis and subcutis. Scale bar represents 400 μm. CD, cluster of differnatiation; *Mitf*, melanogenesis associated transcription factor.
(TIF)

S3 Fig Additional images of the biological replicates described in Fig 6. **(A)** Tg(Dct-Sox10)/Tg(Dct-Sox10); *Mitf*^*mi-vga9/+*^ animals. **(B)** Tg(Dct-Sox10)/Tg(Dct-Sox10); *Mitf*^*mi-vga9/+*^; *Ifih* ^*tm1*.*1Cln/+*^ animals. *Ifih1*, interferon induced with helicase C domain 1; *Mitf*, melanogenesis associated transcription factor.
(TIF)

S4 Fig Immunofluorescent staining of mid-anagen skin isolated from Tg(Dct-Sox10)/0 animals injected with vehicle (water) or 100 μg of poly(I:C), as described in Fig 7. **(A–B)** Skins were double labeled for DCT (red) and CD3ε (green) or DCT and CC3 (green). Nuclei are stained with DAPI (blue). Brackets and arrows indicate the region of the hair bulge and hair bulb, respectively. **(C)** Reactivity of the CC3 antibody was confirmed by staining late catagen hairs that exhibit apoptotic cells within the regressing hair (arrowheads). CC3, cleaved caspase 3; CD, cluster of differentiation; DCT, dopachrome tautomerase; poly(I:C), polyinosinic:polycytidylic acid.
(TIF)

## Abbreviations

Actb: actin beta
CC3: cleaved caspase 3
CD: cluster of differentiation
ChIP: chromatin immunoprecipitation
ChIP-qPCR: chromatin immunoprecipitation quantitative polymerase chain reaction
ChIP-seq: chromatin immunoprecipitation sequencing
DAMP: damage-associated molecular pattern
DCT: dopachrome tautomerase
DEG: differentially expressed gene
FACS: fluorescence-activated cell sorting
IFN: interferon
IgG: immunoglobulin G
Ifih1: interferon induced with helicase C domain 1
IRF: interferon regulatory factor
ISG: IFN-stimulated gene
Mc1r: melanocortin 1 receptor gene
McSC: melanocyte stem cell
Mitf: melanogenesis associated transcription factor
MDA5: melanoma differentiation-associated gene 5
MuLV: murine leukemia virus; mm9, mus musculus 9 genome build
NLRP1: NLR family pyrin domain containing 1
P: postnatal day
PAMP: pathogen-associated molecular pattern
poly(I:C): polyinosinic:polycytidylic acid
PRR: pattern recognition receptor
qRT-PCR: quantitative reverse transcriptase polymerase chain reaction
RNA-seq: RNA sequencing
SG: sebaceous gland
siNC1: negative control siRNA
siRNA: small interfering RNA
SOX10: sex determining region Y-box 10
TICAM1: toll like receptor adaptor molecule 1
