# Supplementary figures and images for: A direct link between MITF, innate immunity, and hair graying
Source: PLoS Biol. 2018 May 3;16(5):e2003648. doi: 10.1371/journal.pbio.2003648 (PMC5933715; doi:10.1371/journal.pbio.2003648)

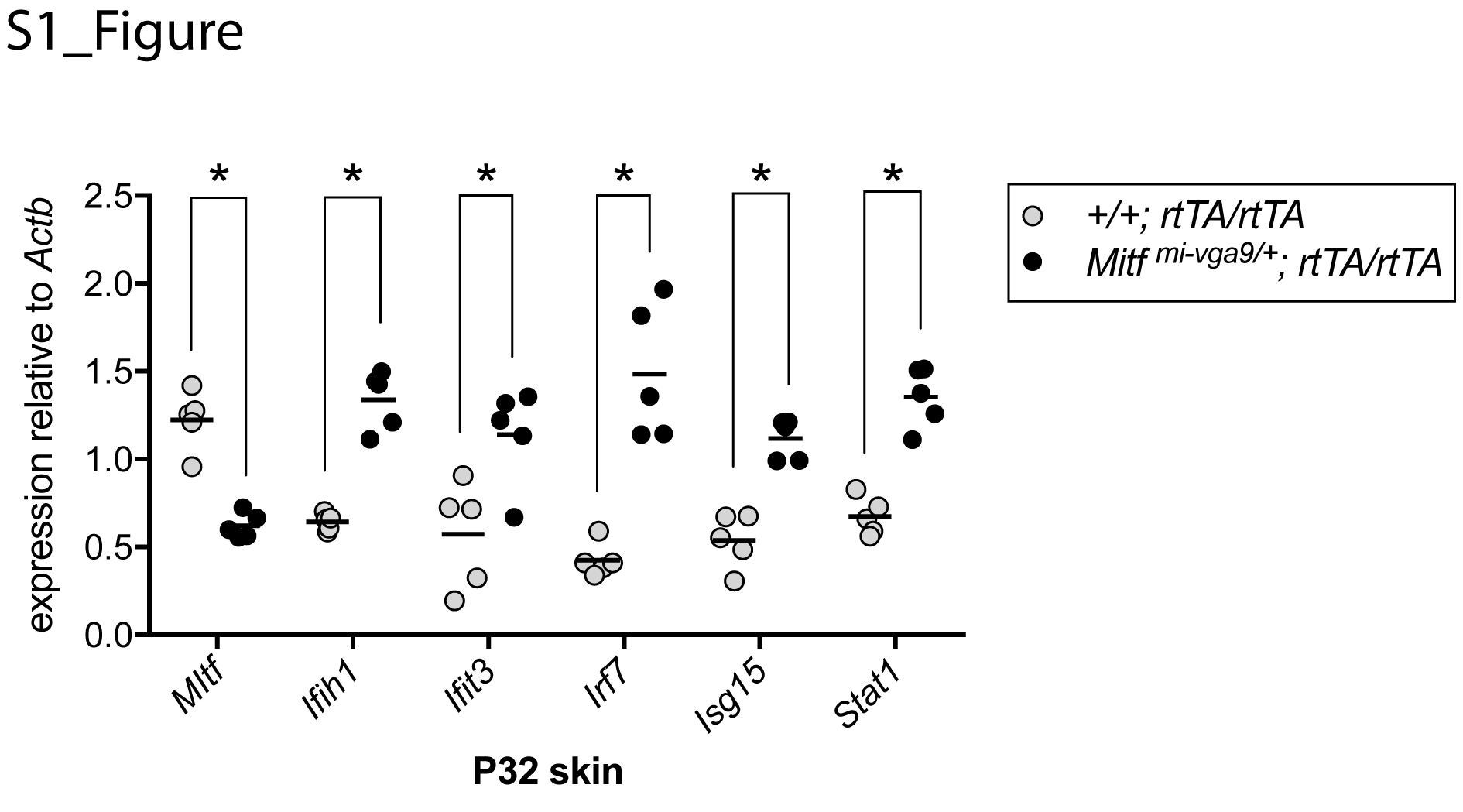

Supplement: S1 Fig — These mice were housed in the animal facility independent from those where the remainder of this study was performed. Each point on the graph represents the expression value from skin of an individual animal. The horizontal bars represent the mean, and the asterisks indicate gene expression changes with a q-value of <0.05 using the two-stage linear step-up procedure of Benjamini, Krieger and Yekutieli, with Q = 5%. ISG, interferon stimulated gene; Mitf, melanogenesis associated transcription factor; P, postnatal day; qRT-PCR, quantitative reverse transcriptase polymerase chain reaction; rtTA, reverse tetracycline-controlled transactivator. (TIF) [file pbio.2003648.s005.tif]

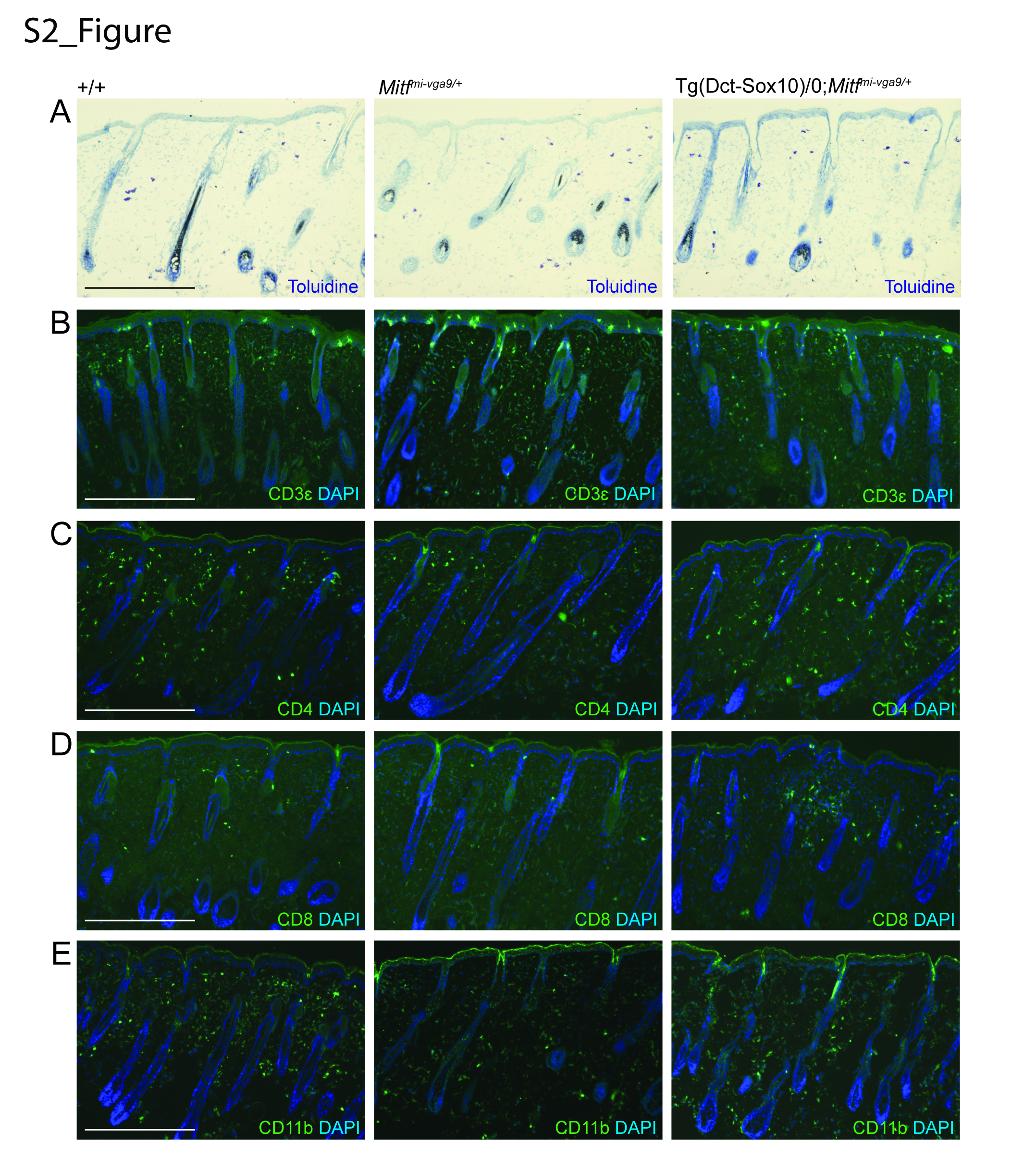

Supplement: S2 Fig — (A) Mast cells were detected using toluidine blue and were found dispersed throughout the dermis. (B–D) Antibodies to CD3ɛ, CD4, and CD8 were used to identify T cells within the epidermis and the dermis. (E) Antibodies against CD11b were used to detect macrophages and Langerhan’s cells and these were distributed within dermis and subcutis. Scale bar represents 400 μm. CD, cluster of differnatiation; Mitf, melanogenesis associated transcription factor. (TIF) [file pbio.2003648.s006.tif]

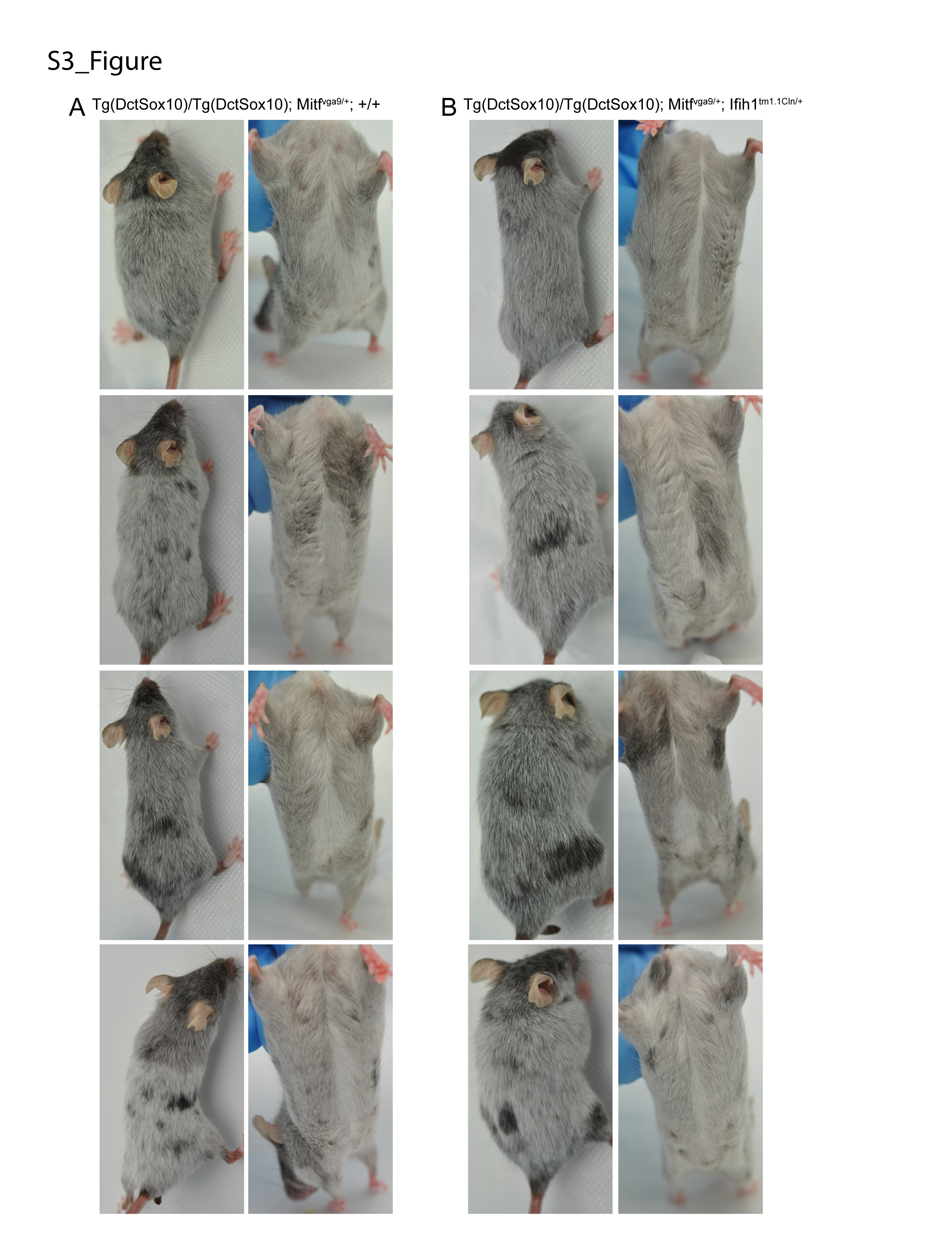

Supplement: S3 Fig — (A) Tg(Dct-Sox10)/Tg(Dct-Sox10); Mitfmi-vga9/+ animals. (B) Tg(Dct-Sox10)/Tg(Dct-Sox10); Mitfmi-vga9/+; Ifih tm1.1Cln/+ animals. Ifih1, interferon induced with helicase C domain 1; Mitf, melanogenesis associated transcription factor. (TIF) [file pbio.2003648.s007.tif]

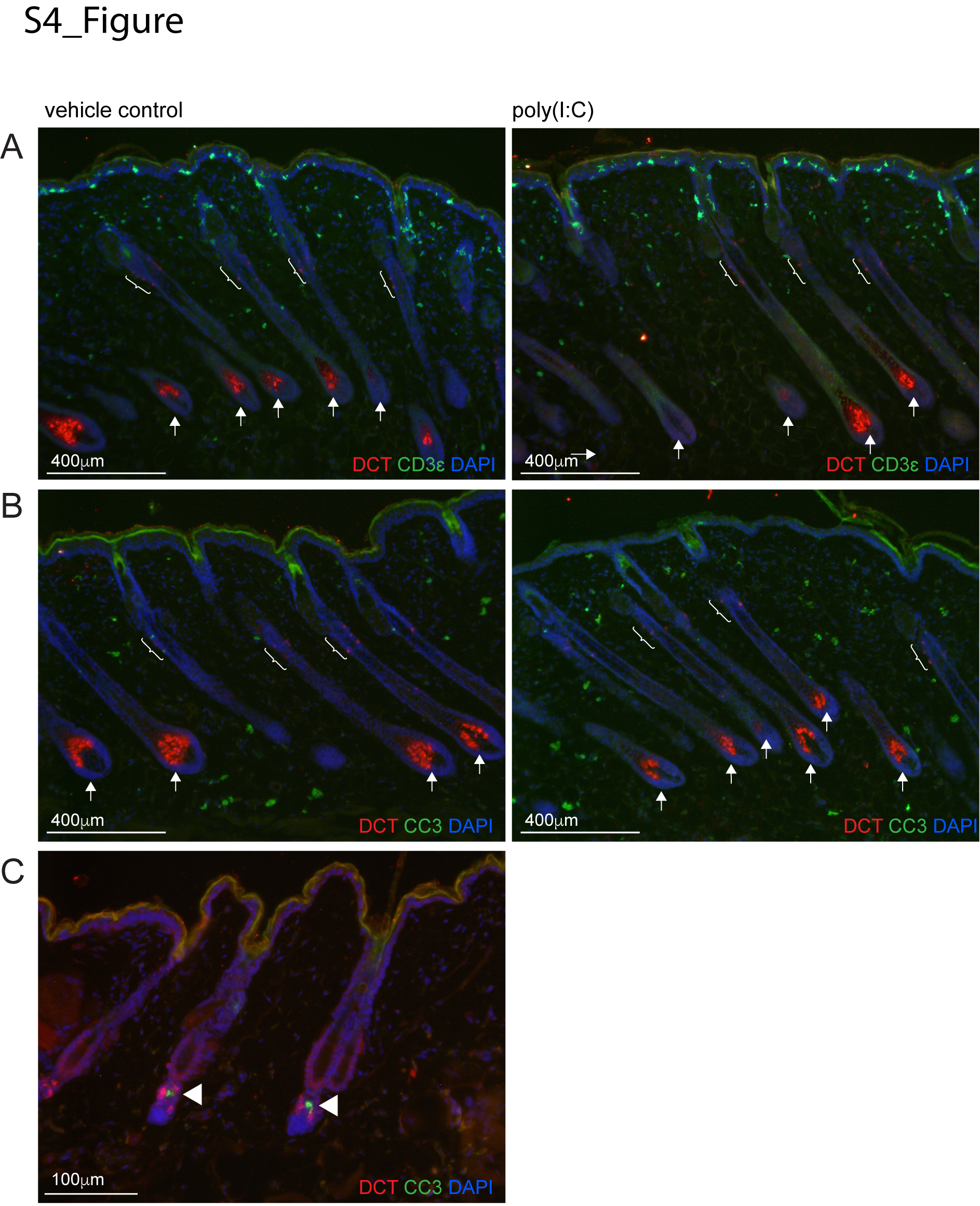

Supplement: S4 Fig — (A–B) Skins were double labeled for DCT (red) and CD3ε (green) or DCT and CC3 (green). Nuclei are stained with DAPI (blue). Brackets and arrows indicate the region of the hair bulge and hair bulb, respectively. (C) Reactivity of the CC3 antibody was confirmed by staining late catagen hairs that exhibit apoptotic cells within the regressing hair (arrowheads). CC3, cleaved caspase 3; CD, cluster of differentiation; DCT, dopachrome tautomerase; poly(I:C), polyinosinic:polycytidylic acid. (TIF) [file pbio.2003648.s008.tif]
